# Multicenter study provides radiomic and biological insights into neoadjuvant chemotherapy response and prognosis in luminal breast cancer

**DOI:** 10.1186/s40644-026-00994-1

**Published:** 2026-02-02

**Authors:** Shiyun Sun, Yansong Bai, Yingnan Bai, Yingying Ding, Yu Xie, Jinlong Zheng, Jiayin Zhou, Tingting Jiang, Yajia Gu, Zhuolin Li, Chao You

**Affiliations:** 1 Department of Radiology, Fudan University Shanghai Cancer Center, Shanghai, 200032 China; 2 Department of Oncology, Shanghai Medical College, Fudan University, No. 270 Dongan Road, Shanghai, 200032 China; 3 Fudan University Institute of Science and Technology for Brain-inspired Intelligence, Shanghai, 200433 China; 4 Department of Radiology, The Third Affiliated Hospital of Kunming Medical University: Yunnan Cancer Hospital, Kunming, China

**Keywords:** Luminal breast cancer, MRI, Radiomics, Heterogeneity, NAC

## Abstract

**Objective:**

Luminal breast cancer shows limited sensitivity to neoadjuvant chemotherapy (NAC) and substantial risk of late recurrence among non-pCR patients. Accurate tools to predict both NAC response and long-term prognosis are urgently needed.

**Materials and methods:**

We retrospectively analyzed 850 patients from three cohorts (FUSCC, YNCC,tures were used for pCR prediction with XGBoost, whereas pre-, post-, and delta (Δ) features informed prognostic modeling with Cox-XGBoost. Performance was evaluated by AUC and C-index with internal and external validation. Independent value of the radiomics-derived RadScore was tested by I-SPY2). A subregion-aware, multitemporal radiomics model was built from DCE- and DWI-MRI, complemented by conventional MRI descriptors (e.g., breast edema, shrinkage pattern) and clinicopathologic variables. Pre-NAC feamultivariable regression. Radiogenomic analyses explored biological underpinnings.

**Results:**

For response prediction, 253 patients from FUSCC (pCR 38/253) and 222 from YNCC (pCR 34/222) were included. Seven radiomics features were retained, mainly from high-perfusion subregions (4/7). The combined model achieved the best performance across cohorts, surpassing radiomics, traditional MRI, and clinical models, with AUCs of 0.83 [0.78-0.88], 0.78 [0.73-0.83], 0.63 [0.57-0.69] and 0.61 [0.55-0.67] in the validation cohort. RadScore derived from the radiomics remained an independent predictor of pCR after adjusting for clinical and MRI variables (OR = 2.06 [1.28–2.15]; *P* = 0.001). For prognosis, 318 non-pCR patients with ≥ 5 years follow-up were analyzed (FUSCC *n* = 160, 44 events; YNCC *n* = 158, 48 events). Nine radiomics features were retained, dominated by delta (Δ) features (5/9) and moderate-perfusion subregions (4/9). The combined model showed the highest prognostic discrimination outperforming radiomics, traditional MRI and clinical models in the validation cohort (0.84 [0.73-0.90] vs 0.81 [0.61, 0.84], 0.60 [0.73-0.90], 0.58 [0.47, 0.69]). Prognostic RadScore remained independentlnedy associated with recurrence (HR 4.78, 95% CI 2.54–5.89; *P* = 0.001), along with post-NAC Ki-67, diffuse edema, and non-concentric shrinkage. Radiogenomic validation confirmed that high-perfusion features driving response were enriched in drug-metabolism, PI3K-Akt, and estrogen signaling pathways, whereas moderate-perfusion delta (Δ) features driving prognosis aligned with hypoxia and immune-evasion programs associated with recurrence.

**Conclusion:**

Subregion-aware, multitemporal radiomics accurately and interpretabily predicts NAC response and long-term prognosis in luminal breast cancer, supporting individualized treatment selection and risk stratification.

**Supplementary Information:**

The online version contains supplementary material available at 10.1186/s40644-026-00994-1.

## Introduction

Luminal breast cancer constitutes the largest breast cancer subtype but remains a therapeutic challenge in the neoadjuvant setting [1]. While NAC is routinely used to downstage locally advanced tumors or enable breast conservation, luminal cancers respond poorly compared with HR-negative subtypes, with reported pCR rates as low as 7–15% [2, 3]. This limited chemosensitivity underscores the need to identify, prior to treatment, which patients are likely to benefit from NAC to avoid unnecessary toxicity. Equally important, non-pCR patients continue to face substantial risks of late recurrence, often extending beyond five years, highlighting the need for accurate post-treatment prognostication [4]. Together, these challenges point to a critical unmet need for biomarkers that can inform both pre-treatment selection and long-term risk stratification in luminal breast cancer.

Radiomics and artificial intelligence (AI) have shown promise for predicting NAC outcomes by quantifying intratumoral heterogeneity on MRI [5–8]. Early studies reported associations between pretreatment heterogeneity indices and treatment response or recurrence, and more recent multimodal or spatiotemporal approaches have further improved predictive accuracy in multicenter settings. However, most existing models were trained across mixed breast cancer subtypes and provide limited biological interpretability, leaving uncertainty about their applicability in luminal disease. Furthermore, although radiogenomic analyses have begun to link imaging features with gene expression programs, such efforts have not been specifically contextualized in luminal breast cancer [9, 10].

In this study, we aimed to address this gap by developing and validating luminal-specific models for predicting both NAC response and long-term prognosis. Building on DCE- and DWI-derived multiparametric MRI, we applied a subregion-aware, multitemporal radiomics approach, complemented by refined conventional MRI and clinicopathologic features, and validated the models across independent cohorts with long-term follow-up. To improve interpretability, we performed radiogenomic analyses to provide luminal-specific, interpretable models that support individualized decision-making for NAC selection and post-treatment risk stratification.

## Methods

### Patients

**Fig. 1:**
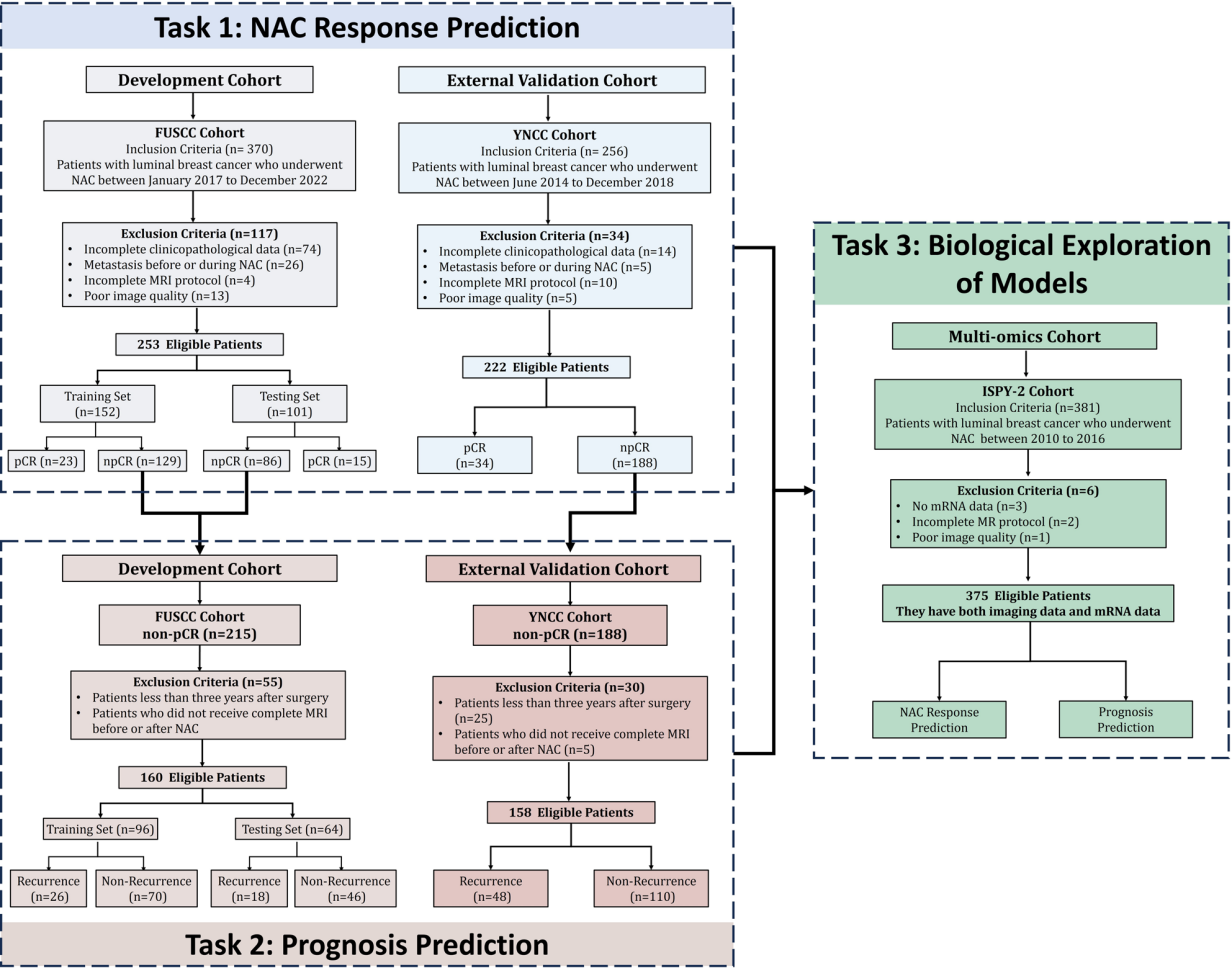
A total of 253 and 222 eligible patients with luminal breast cancer from the FUSCC and YNCC cohorts, respectively, were included for NAC response prediction (Task 1). For prognosis prediction (Task 2), 160 non-pCR patients from FUSCC and 158 from YNCC with complete pre- and post-NAC MRI were analyzed. For biological exploration (Task 3), 375 patients with luminal breast cancer from the I-SPY2 cohort with both MRI and mRNA data were included to investigate the molecular basis of prediction model.

This multicenter retrospective study was approved by the Institutional Review Board of Fudan University Shanghai Cancer Center (approval No. 2402291-2), with informed consent waived. Patients with luminal breast cancer (HR + and HER2–) who underwent standard NAC were consecutively enrolled from three independent cohorts: FUSCC (2017–2022), YNCC (2014–2018), and the I-SPY2 dataset (2010–2016) (Fig. 1).

Inclusion criteria were: (1) histologically confirmed luminal breast cancer; (2) availability of pretreatment contrast-enhanced MRI; and (3) complete clinicopathological and follow-up records. Exclusion criteria were: (1) distant metastasis; (2) incomplete MRI protocol; (3) poor image quality; and (4) incomplete clinical data. For prognosis analysis, only patients with ≥ 5 years of follow-up were included to account for late recurrence. Baseline characteristics are summarized in Table 1.

**Table 1:** Summary of demographic and clinicopathologic data from three cohorts

	FUSCC Cohort (*n* = 253)	YNCC Cohort (*n* = 222)	I-SPY 2 Cohort (*n* = 375)
**Age (years)**	47 (21–67)	45 (25–77)	NA
**Menopause status**			NA
Menopausal	134 (52.96)	124 (55.86)	
Premenopausal	119 (47.04)	98 (44.14)	
**T stage**			NA
cT1-2	46 (18.18)	34 (15.32)	
cT3-4	207 (81.82)	188 (84.68)	
**N stage**			NA
cN0-1	51 (20.16)	40 (18.02)	
cN2-3	202 (79.84)	182 (81.98)	
**ER status**			NA
Positive	235 (92.89)	204 (91.89)	
Negative	18 (7.11)	18 (8.11)	
**PR status**			NA
Positive	198 (78.26)	182 (81.98)	
Negative	55 (21.74)	40 (18.02)	
**Ki67 index**			NA
≥ 20%	41 (16.22)	38 (17.12)	
< 20%	212 (83.79)	184 (82.88)	
**Subtype**			NA
Luminal A	172 (67.98)	141 (63.51)	
Luminal B	81 (32.02)	81 (36.49)	
**Neoadjuvant chemotherapy scheme**			
Anthracycline-cyclophosphamide	65 (25.69)	66 (29.73)	0
Anthracycline-cyclophosphamide with Paclitaxel	188 (74.31)	187 (70.27)	94 (25.07)
Other#	0	0	281 (74.93)
**pCR**			
Yes	38 (15.02)	34 (15.32)	64 (17.07)
No	215 (84.98)	188 (84.68)	311 (82.93)
**Recurrence**	*N* = 160	*N* = 158	NA
Event	44 (27.50)	48 (30.38)	
No Event	116 (72.50)	110 (69.62)	
**DFS (years)**	4.75(0.69–6.53)	5.28 (0.75–9.88)	NA

*For continuous variables are median and interquartile range M (IQR); for categorical variables are number and percentage N (%). ER: estrogen receptor. PR: progesterone receptor. NA: not applicable

pCR: Pathological complete response. DFS: Disease-free survival

# I-SPY2 tested several novel paclitaxel-based regimens; in this study it was used exclusively as a biology-focused exploratory cohort, so regimen heterogeneity does not influence biological analysis

### Study design

**Fig. 2:**
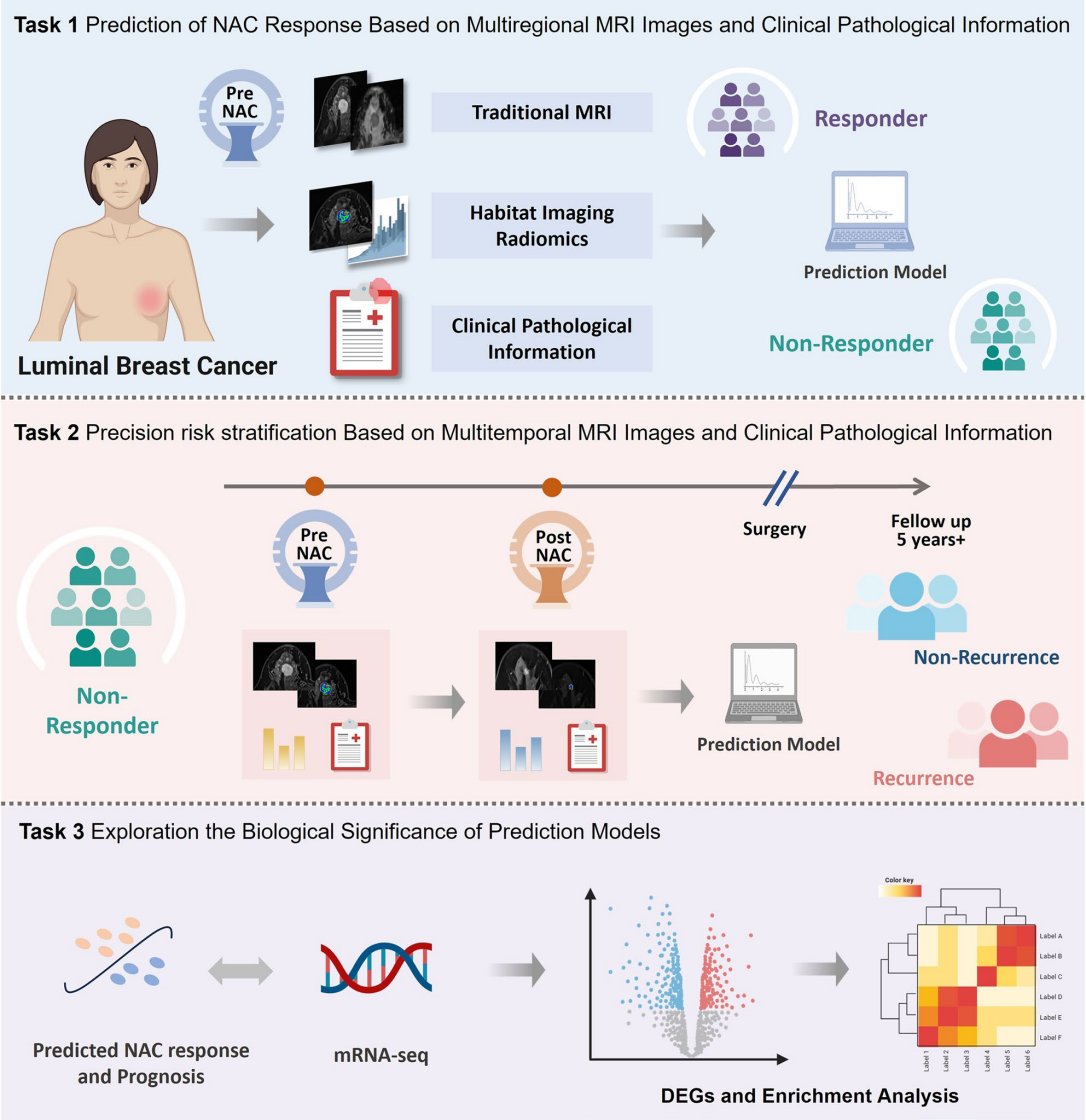
Schematic overview of the study design. Task 1 involved prediction of NAC response in luminal breast cancer using pre-treatment multiparametric MRI (traditional MRI features, subregion-based radiomics) combined with clinicopathologic information. Task 2 focused on prognosis modeling among non-pCR patients, incorporating pre- and post-NAC MRI and clinical data to stratify long-term recurrence risk over more than 5 years of follow-up. Task 3 explored the biological significance of the prediction models by integrating imaging-derived RadScores with mRNA sequencing data for differential gene expression and pathway enrichment analyses.

**Fig. 3:**
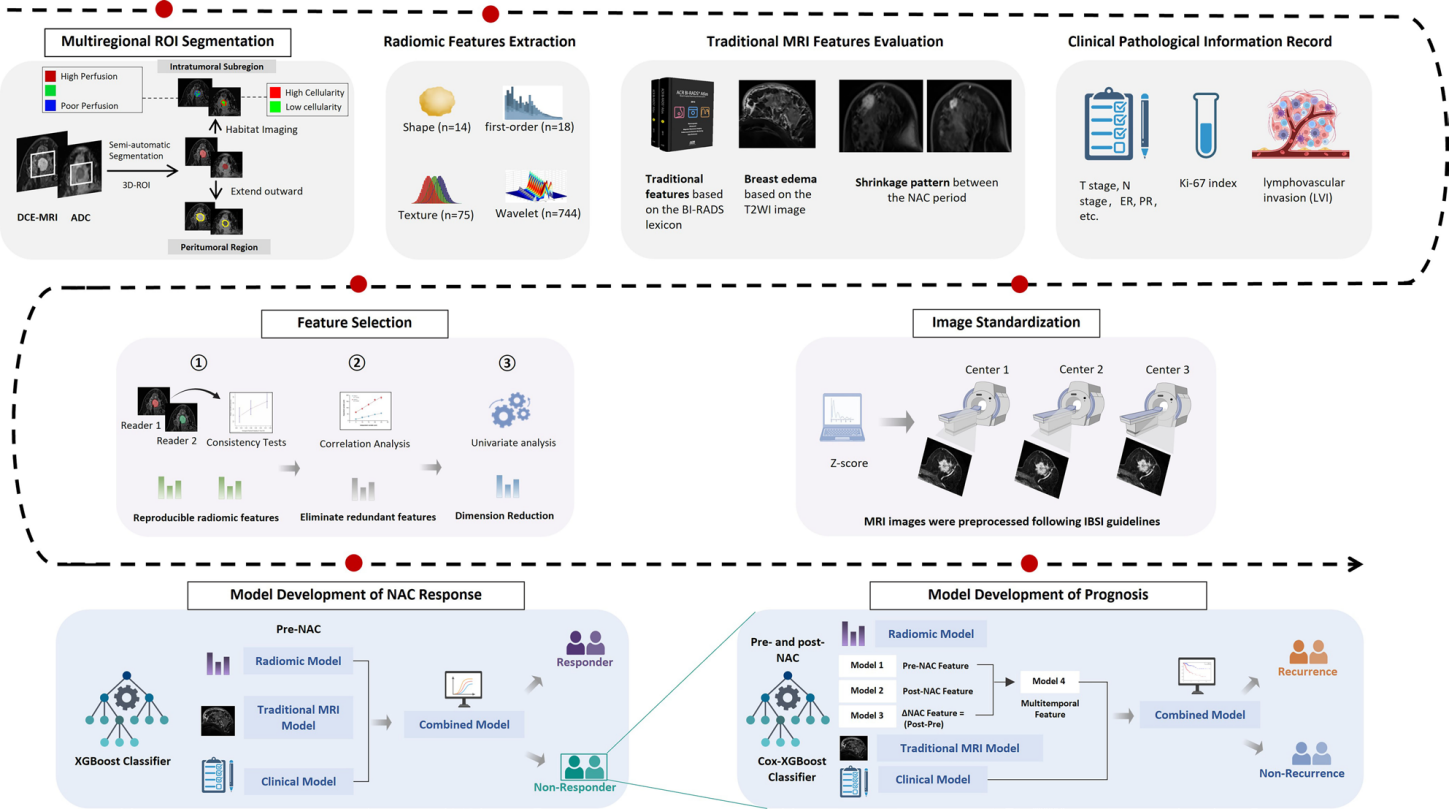
Workflow of image analysis and model development. Intratumoral subregions (high-, moderate-, and poor-perfusion; high- and low-cellularity) and peritumoral regions were segmented on DCE-MRI and ADC maps. Radiomic features were extracted alongside conventional MRI features (e.g., breast edema, shrinkage pattern) and clinicopathologic variables. Images were standardized across centers, and features underwent reproducibility testing, redundancy removal, and univariate analysis for dimensionality reduction. Response prediction models were built on pre-NAC features using XGBoost, while prognostic models incorporated pre-, post-, and delta features using Cox-XGBoost. Combined models integrating radiomic, traditional MRI, and clinical features were constructed for both tasks.

The overall study design is depicted in Figs. 2 and 3. Radiomic, traditional MRI, and clinical features were extracted from both pre-treatment and post-treatment images to characterize tumor heterogeneity across spatial and temporal dimensions. Pretreatment features were used to establish a prediction model for pCR, whereas delta features derived from paired pre- and post-NAC imaging were employed to construct a prognostic model for DFS. To further elucidate the biological relevance of key radiomic features, transcriptomic data from the I-SPY1 cohort were integrated through gene expression analysis.

### Clinical outcomes

Treatment response was defined as pathological complete response (pCR), i.e., no residual invasive carcinoma in the breast or axillary nodes (ypT0/is ypN0) following NAC. Prognosis was evaluated using disease-free survival (DFS), measured from diagnosis to first recurrence, contralateral breast cancer, or death. Patients without events were censored at last follow-up. For prognostic analysis, only patients with ≥ 5 years of potential follow-up were included to account for late recurrence, with longer follow-up incorporated when available. Overall survival was not analyzed due to limited long-term data in some cohorts.

### MRI acquisition and preprocessing

MRI was performed on 1.5-T scanners with dedicated breast coils, including T1WI, T2WI, DWI, and DCE-MRI. Detailed parameters are in the Supplementary File [11]. To reduce inter-center variability, images were resampled to isotropic 1 × 1 × 1 mm³ using B-spline interpolation, z-score normalized, and ComBat-harmonized per IBSI recommendations [11].

### Tumor segmentation

Tumor segmentation was performed in 3D Slicer (version 5.6.1). Two board-certified breast radiologists (8 and 12 years of experience), blinded to outcomes, independently delineated the whole tumor on the first post-contrast DCE-MRI and ADC maps using a semi-automatic method. The peritumoral region was generated by expanding the tumor boundary outward 5 mm and subtracting the tumor volume.

For intratumoral subregions, modality-specific strategies were applied. On DCE-MRI, k-means clustering of four kinetic parameters—wash-in slope (WIS), wash-out slope (WOS), signal enhancement ratio (SER), and percentage enhancement (PE)—partitioned tumors into high-, moderate-, and poor-perfusion regions (Figure E1–E2) [6]. On DWI, voxels were stratified into high- and low-cellularity areas using mean ADC thresholding (Figure E3).

To assess reproducibility, each radiologist segmented 50 randomly selected tumors. Radiomic features were extracted from these ROIs, and intraclass correlation coefficients (ICCs) were calculated; features with ICC ≥ 0.80 were considered stable. Given the high reproducibility, ROIs from the more experienced radiologist were used for final analysis.

### Radiomic feature extraction

Radiomic analysis was performed using the open-source PyRadiomics package (version 3.0, Python 3.6). From each ROI, a total of 873 features were initially extracted, including 14 shape features, 18 first-order statistics, 75 texture features derived from gray-level matrices [gray-level co-occurrence matrix (GLCM), gray-level run-length matrix (GLRLM), gray-level size zone matrix (GLSZM), gray-level dependence matrix (GLDM), and neighboring gray-tone difference matrix (NGTDM)] and wavelet-based features (Supplementary Table E1).

For treatment response prediction, only features extracted from pre-NAC images were analyzed. For prognosis prediction, both pre- and post-NAC features were extracted, and delta features were computed to quantify temporal heterogeneity using the formula:

$$\eqalign{ \Delta & Feature \cr & = Featur{e_{post - NAC}} - Featur{e_{pre - NAC}} \cr}$$

All features were extracted in compliance with IBSI guidelines, with image preprocessing (resampling, discretization, normalization) standardized across cohorts.

### Traditional MRI features

Traditional MRI features before and after NAC were evaluated according to the Breast Imaging Reporting and Data System (BI-RADS) lexicon, including tumor shape, margins, internal enhancement pattern, background parenchymal enhancement (BPE) [12]. In addition, breast edema (peritumoral, subcutaneous or subcutaneous or prepectoral, and diffuse edema) and shrinkage pattern (concentric and non-concentric shrinkage) also be evaluated according to our previous study [13]. These features were independently recorded by the same two radiologists who performed segmentation (blinded to outcomes), with discrepancies resolved by consensus (Supplementary Table E2).

### Clinicopathologic variables

Clinicopathologic variables included clinical T and N stage, estrogen receptor (ER) and progesterone receptor (PR) expression, HER2 status, Ki-67 index, and lymphovascular invasion (LVI). Patients were categorized as luminal A or luminal B using immunohistochemical surrogates, and standard NAC regimens (anthracycline–cyclophosphamide with or without paclitaxel) were applied.

### Feature selection and model development

All extracted features were standardized using z-score transformation. To ensure reproducibility, only features with ICC ≥ 0.80 were retained, and highly correlated features (Spearman’s ρ ≥ 0.9) were excluded. For treatment response prediction, pre-NAC features were first reduced using the Wilcoxon rank-sum test (*p* < 0.05). To further control overfitting and enhance interpretability, we adopted an XGBoost-embedded feature-shrinkage strategy with nested cross-validation and stability selection: within the development cohort, models ranked features by gain in repeated inner resampling and retained those consistently selected across resamples (frequency threshold prespecified). Traditional MRI descriptors and clinicopathologic covariates were screened by univariable analyses (logistic for pCR; Cox for DFS), and variables with *P* < 0.10 together with a priori covariates were retained for multivariable modeling.

For prognosis, radiomic features from pre-, post-, and delta images were evaluated using the same framework, applying a Cox-XGBoost objective and selecting stable features by inner resampling while optimizing Harrell’s C-index. The resulting model outputs (RadScores) were combined with clinicoradiologic variables to build integrated models. Class imbalance for pCR was addressed with SMOTE. Performance was assessed by AUC (pCR) and C-index (DFS), with 95% CIs estimated by 1,000 bootstrap resamples. Internal evaluation used nested cross-validation in the development cohort, and external validation was performed in an independent cohort to assess generalizability and mitigate overfitting.

### Biological analysis

To explore the biological underpinnings of radiomic heterogeneity, transcriptomic validation was performed in the I-SPY2 cohort (*n* = 375) providing pretreatment DCE-MRI, ADC maps, and matched mRNA profiles. Patients were stratified into high- and low-risk groups using RadScores from the final response and prognostic radiomic models. The cutoff, determined by maximizing the Youden index and C-index in the training cohort, was then applied to I-SPY2 for consistent risk stratification. Differentially expressed genes (DEGs) were identified with limma (empirical Bayes; |log₂FC| ≥2, BH FDR < 0.05) [15]. Functional enrichment was performed by KEGG over-representation analysis with BH correction (q < 0.05) [16]. Immune cell infiltration was estimated by ssGSEA, and between-group differences tested by Wilcoxon rank-sum with BH adjustment [16].

### Statistical analysis

All analyses were performed using R software (version 4.3.0). Continuous variables were compared using the Wilcoxon rank-sum test, and categorical variables using the χ² or Fisher’s exact test. Survival outcomes were analyzed with the Kaplan–Meier method and compared by the log-rank test. To further assess independent predictive value, the radiomic score (RadScore) derived from the model outputs was incorporated into multivariable logistic regression for pCR and Cox proportional hazards regression for DFS together with clinicopathologic variables. A two-sided *p* < 0.05 was considered statistically significant.

## Results

### Patient characteristics

A total of 850 patients with luminal breast cancer were included in this study. For response prediction, 253 patients from the FUSCC cohort were used, including 152 in the training set and 101 in the internal testing set. The external validation cohort comprised 222 patients from YNCC. The pCR rates were 15.0% in FUSCC and 15.3% in YNCC. The I-SPY2 cohort (*n* = 375) was not used for model training or validation but served exclusively for transcriptomic analysis (Table 1). For prognostic prediction, 318 patients with at least five years of follow-up were included: 160 from FUSCC and 158 from YNCC. Recurrence events occurred in 44 (27.5%) and 48 (30.4%) patients, respectively, with median follow-up times of 5.8 years (range, 5.0-7.5) in FUSCC and 6.1 years (range, 5.7–6.6) in YNCC.

### Prediction of NAC response

#### Radiomic feature selection and importance

**Fig. 4:**
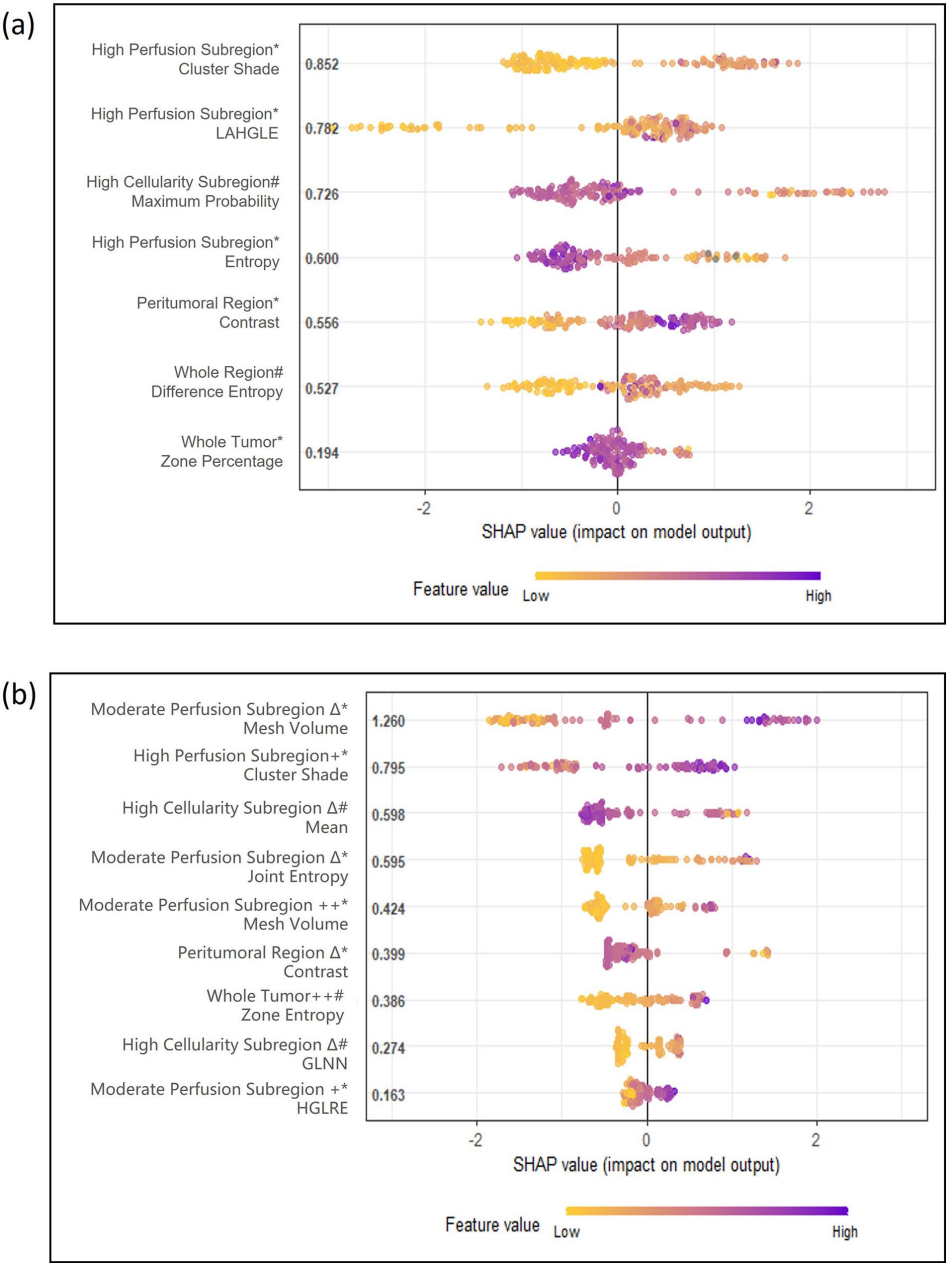
Feature importance in response and prognostic models. SHAP summary plots show the top contributing features for (**a**) the NAC response classifier and (**b**) the prognostic model. Each dot represents one patient; colors indicate feature values (yellow = low, purple = high), and the horizontal position indicates SHAP values that reflect the impact on the model output. High-perfusion subregion features mainly drove response prediction, whereas dynamic changes in moderate-perfusion subregions dominated prognostic modeling.* DCE sequence; # ADC sequence; + pre-NAC; ++ post-NAC; Δ longitudinal change during NAC.

Seven radiomic features were identified as key predictors of pCR in the FUSCC training set (Supplementary Table E3), with their relative contributions illustrated by SHAP values (Fig. 4a). Intratumoral subregion features were most prevalent (4/7), highlighting the strong association between spatial heterogeneity and NAC response, particularly within high-perfusion subregions (HPS) that typically reflect spatial variations in intratumoral microvascular distribution and density.

#### Traditional MRI and clinical predictive factors

In addition, several conventional MRI and clinical variables were independent predictors of pCR, including pre-NAC diffuse edema (odds ratio [OR] = 0.86, 95% CI: 0.33–0.93; *p* = 0.007), T stage (OR = 0.93, 95% CI: 0.46–0.96; *p* = 0.003), and Ki-67 index (OR = 1.34, 95% CI: 1.16–1.71; *p* = 0.005) (Supplementary Table E4).

#### Performance of combined models

**Fig. 5:**
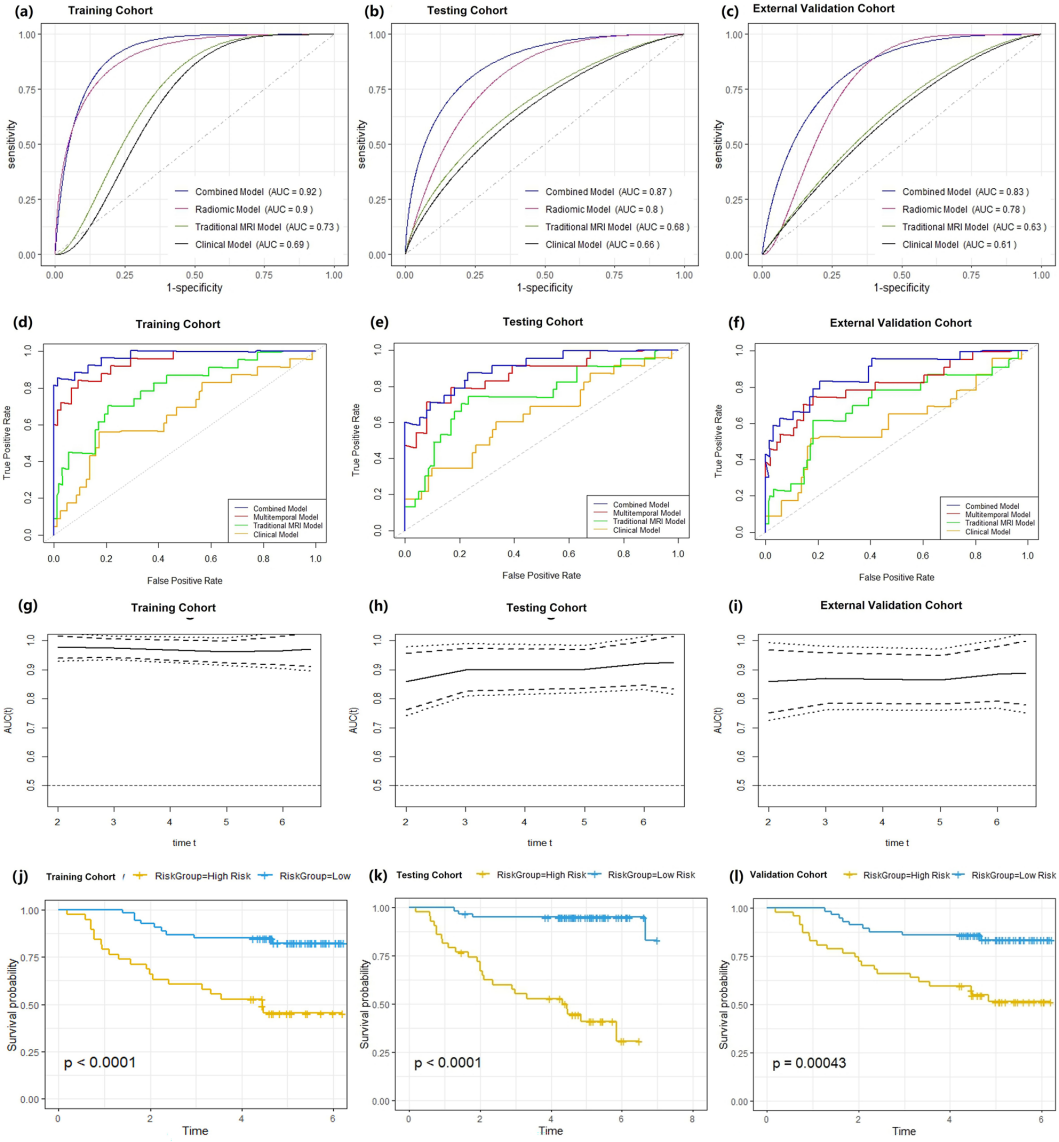
Performance of response and prognostic models. (**a**–**c**) ROC curves of NAC response prediction models in the training, testing, and external validation cohorts. (**d**–**f**) Precision–recall curves in the corresponding cohorts. (**g**–**i**) Time-dependent AUCs of prognostic models for disease-free survival prediction in the training, testing, and external validation cohorts. (**j**–**l**) Kaplan–Meier survival analyses demonstrate significant separation between high- and low-risk groups defined by the prognostic RadScore across all cohorts

**Fig. 6:**
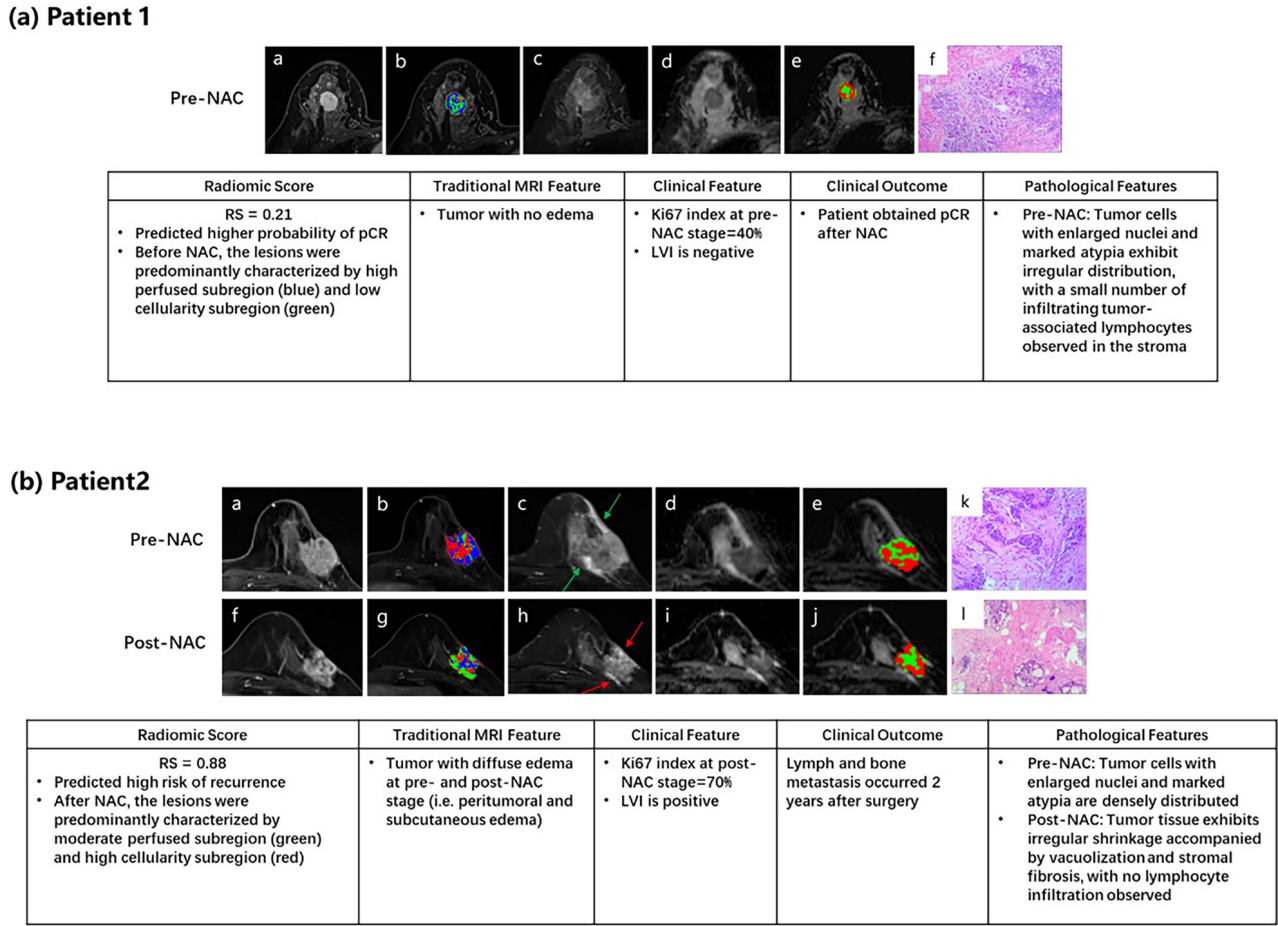
Representative patient cases illustrating model predictions and pathological validation. (**a**) Patient 1 with a low RadScore (0.21) predicted higher probability of pCR. Pre-NAC lesions were predominantly composed of high-perfusion and low-cellularity subregions, with no edema on conventional MRI. The patient achieved pCR after NAC, and pathology confirmed tumor cell atypia with limited lymphocyte infiltration. (**b**) Patient 2 with a high RadScore (0.88) predicted high recurrence risk. Pre- and post-NAC MRI showed diffuse edema, and post-NAC lesions were mainly composed of moderate-perfusion and high-cellularity subregions. The patient developed lymph and bone metastases 2 years after surgery, and pathology revealed irregular shrinkage and stromal fibrosis without lymphocyte infiltration.

The combined model achieved the highest performance across cohorts, outperforming the radiomics, traditional MRI, and clinical models in the FUSCC training set (AUC 0.92 [0.87–0.96] vs. 0.90 [0.85–0.94], 0.73 [0.67–0.79], 0.69 [0.63–0.75]), internal testing set (0.87 [0.82–0.92] vs. 0.80 [0.75–0.85], 0.68 [0.62–0.74], 0.66 [0.60–0.72]), and YNCC cohort (0.83 [0.78–0.88] vs. 0.78 [0.73–0.83], 0.63 [0.57–0.69], 0.61 [0.55–0.67]) (Table 2; Fig. 5a-c). In the combined model framework, the RadScore derived from the radiomics model remained an independent predictor of pCR after adjusting for clinical and MRI variables (OR = 2.06 [1.28–2.15]; *p* = 0.001) (Supplementary Figure E4a). Representative case applications of prognostic models are illustrated in Fig. 6a.

**Table 2:** The performance of prediction models for NAC response and prognosis

AUC (95%)	Training Cohort	Testing Cohort	External Validation Cohort
**NAC Response**			
Combined Model*	0.92 (0.87, 0.96)	0.87 (0.82, 0.92)	0.83 (0.78, 0.88)
**Radiomic Model**	0.90 (0.85, 0.94)	0.80 (0.75, 0.85)	0.78 (0.73, 0.83)
**Traditional MRI Model**	0.73 (0.67, 0.79)	0.68 (0.62, 0.74)	0.63 (0.57, 0.69)
**Clinical Model**	0.69 (0.63, 0.75)	0.66 (0.60, 0.72)	0.61 (0.55, 0.67)
**Prognosis**			
**Combined Model#**	0.95 (0.88, 0.99)	0.87 (0.84, 0.95)	0.84 (0.73, 0.90)
**Radiomics Model**			
Multitemporal Model	0.92 (0.83,0.97)	0.85 (0.77, 0.93)	0.81 (0.61, 0.84)
ΔNAC Model	0.84 (0.79, 0.93)	0.78 (0.56, 0.87)	0.75 (0.61, 0.86)
Post-NAC Model	0.79 (0.71, 0.89)	0.69 (0.60, 0.85)	0.62 (0.51, 0.76)
Pre-NAC Model	0.77 (0.72, 0.88)	0.71 (0.63, 0.81)	0.65 (0.51, 0.71)
**Traditional MRI Model**	0.70 (0.64, 0.76)	0.65 (0.59, 0.71)	0.60 (0.54, 0.66)
**Clinical Model**	0.66 (0.53, 0.79)	0.60 (0.51, 0.70)	0.58 (0.47, 0.69)

The combined model * for predicting response consists of pre-NAC radiomics, traditional MRI and clinical features

The combined model # for predicting prognosis consists of features during NAC, including multitemporal radiomics, traditional MRI and clinical features during NAC

#### Subtype-specific predictive performance

Subgroup analysis by molecular subtype showed consistent predictive performance in both luminal A and luminal B tumors, with AUCs of 0.81 (0.71–0.90) and 0.86 (0.77–0.93) in the training set, and 0.80 (0.69–0.89) and 0.85 (0.74–0.92) in the validation set, respectively (Supplementary Fig. 5a-b).

Together, these findings highlight the importance of intratumoral subregion features that capture spatial heterogeneity for pCR prediction, while also indicating that integration with conventional MRI and clinicopathologic factors can further enhance model performance and clinical applicability.

### Prediction of prognosis

#### Radiomic feature selection and importance

For prognosis prediction in non-pCR patients, models based on pre-, post-, and delta features individually showed moderate discrimination (C-index 0.77 [0.72–0.88], 0.79 [0.71–0.89], 0.84 [0.79–0.93]), whereas the multitemporal radiomics model achieved superior performance (0.92 [0.83–0.97], Table 2; Supplementary Figure E6). In this final model, nine features were retained, with delta features (5/9) and moderate-perfusion subregion (MPS) features (4/9) being most prevalent, reflecting the contribution of both temporal and spatial heterogeneity to prognostic prediction. The volume change of the moderate-perfusion subregion during NAC (delta_dce_mps_MeshVolume) had the greatest impact (SHAP = 1.26), while feature (pre_dce_hps_ClusterShade) also demonstrated prognostic relevance (SHAP = 0.80), serving as a shared predictor of both response and recurrence (Fig. 4b, Table E5).

#### Traditional MRI and clinical prognostic factors

Traditional MRI and clinical factors independently associated with recurrence included post-NAC subcutaneous or prepectoral (HR = 1.16 [1.02–1.33]; *p* = 0.031), diffuse edema (HR = 6.42 [2.33–7.66]; *p* < 0.001), non-concentric shrinkage (HR = 2.85 [1.15–5.07]; *p* = 0.024), higher post-NAC Ki-67 index (HR = 3.13 [1.38–7.10]; *p* = 0.007), and positive LVI (HR = 1.78 [1.04–4.21]; *p* = 0.011) (Supplementary Table E6).

#### Performance of combined models

When integrated with MRI and clinical factors, the combined model showed the highest prognostic discrimination outperforming radiomics, traditional MRI and clinical models in the validation cohort (0.84 [0.73-0.90] vs 0.81 [0.61, 0.84], 0.60 [0.73-0.90], 0.58 [0.47,0.69]) (Table 2, Fig. 5d-f). Time-dependent ROC curves confirmed stable performance, and Kaplan–Meier analysis showed significantly worse DFS in high-risk patients (38.44–44.50% vs. 87.97–91.10% at 5 years; *p* < 0.001; Fig. 4g-l). Importantly, within this framework the RadScore remained independently associated with recurrence after adjustment for conventional prognostic factors (HR = 4.78 [2.54–5.89]; *p* = 0.001; Supplementary Figure E4b). Representative case applications of prognostic models are illustrated in Fig. 6b.

#### Subtype-specific prognostic stratification

Subtype analysis further demonstrated that the combined model could stratify high- and low-risk patients within both luminal A and luminal B groups, with significantly different DFS by Kaplan-Meier analysis (all *p* < 0.0001; Supplementary Figure E5c-f).

In conclusion, the multitemporal radiomic model incorporating spatiotemporal information accurately predicted recurrence risk in non-pCR patients, and its integration with MRI and clinical features further improved robustness and clinical applicability.

### Biological validation

**Fig. 7:**
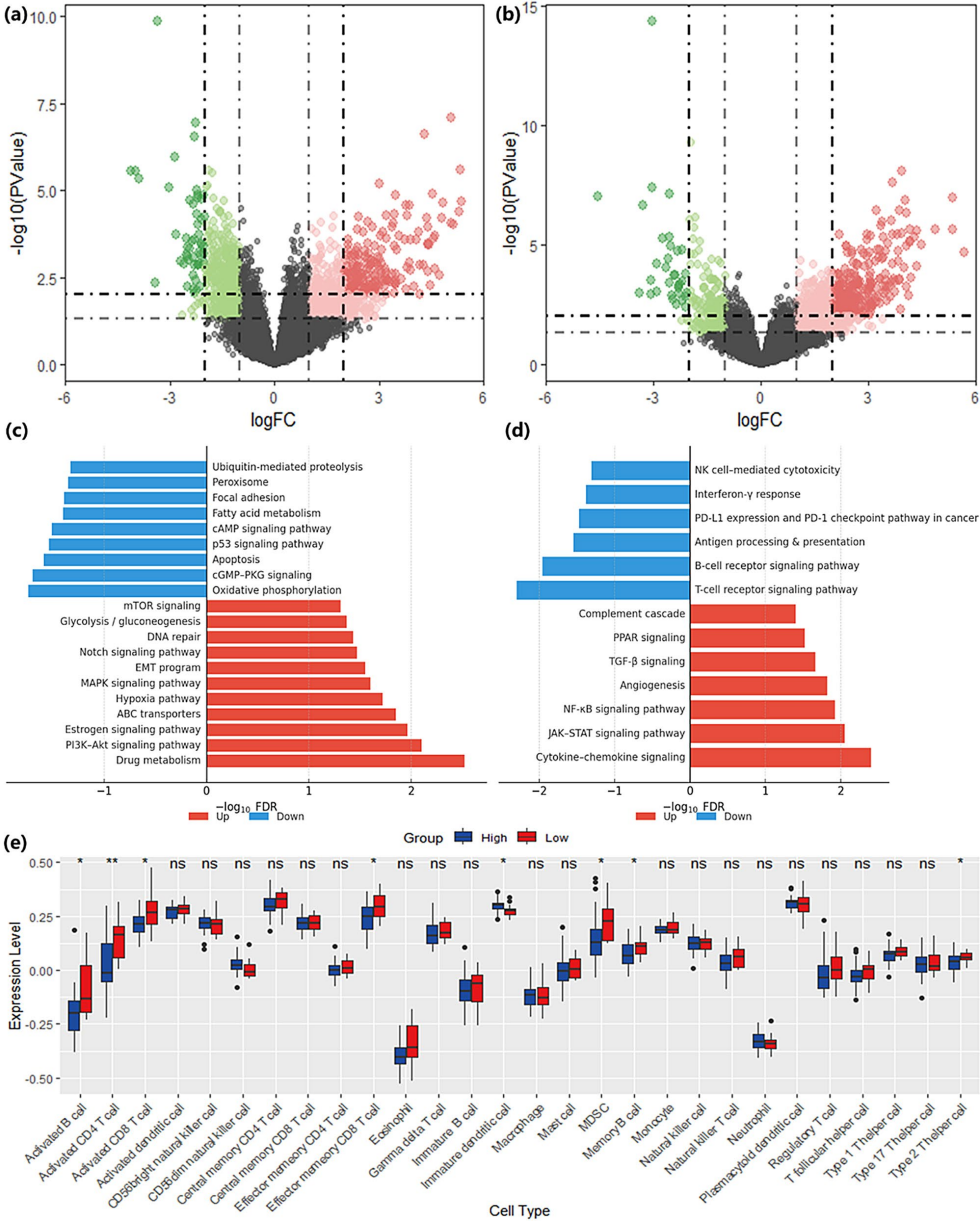
Radiogenomic analyses of prediction models. (**a**–**b**) Volcano plots of differentially expressed genes (DEGs) between high- and low-risk groups defined by response (**a**) and prognostic (**b**) RadScores. (**c**–**d**) KEGG pathway enrichment analyses of DEGs highlight activation of estrogen signaling, PI3K–Akt/mTOR, and cell-cycle/RTK pathways in non-pCR groups (**c**), and hypoxia- and immunosuppression-related pathways in high-risk prognostic groups (**d**). (**e**) Comparison of immune cell infiltration between high- and low-risk groups shows modest but consistent differences, with suppressed immune activity observed in high-risk patients

Using the pre-specified cutoffs from the training cohort, I-SPY2 patients (*n* = 375) were dichotomized into high- and low-risk groups for the response (non-pCR propensity) and prognosis (recurrence risk) analyses, respectively. Differential expression analysis revealed substantial transcriptomic differences between groups (FDR < 0.05, Fig. 7a-b), supporting that radiomic risk stratification reflects underlying biology (Supplementary Table E7).

### Response-related biological validation

Compared with the low-risk group, the high-risk (non-pCR-prone) group showed significant enrichment of pathways related to drug metabolism, PI3K-Akt signaling, and estrogen signaling, alongside relative down-regulation of intracellular signaling cascades such as cGMP-PKG and cAMP (GSEA, FDR < 0.05; Fig. 7c, Table E8). Explainability analysis highlighted high-perfusion subregion features as the dominant contributors to the response classifier, underscoring spatially prominent HPS as a key imaging correlate of NAC resistance; within this context, the GLCM ClusterShade from HPS emerged as a shared predictor of both response and recurrence, implicating greater enhancement asymmetry-indicative of pronounced spatial heterogeneity and microvascular complexity-in reduced chemosensitivity and elevated relapse risk. These enrichments remained significant after FDR control within the prespecified family, with a limited subset passing q < 0.05.

### Prognosis- related biological validation

The high-recurrence-risk group exhibited enrichment of cytokine/chemokine signaling, JAK–STAT, and PPAR pathways, accompanied by suppression of immune-related programs (T-cell receptor, B-cell receptor, PD-1/PD-L1) on GSEA (FDR < 0.05; Fig. 7d, Table E9). ssGSEA further confirmed that only a small subset of immune populations differed between the high- and low-risk groups—primarily CD8⁺ T cells and B cells—consistent with a low-immune milieu in the high-risk group (Fig. 7e). Moderate-perfusion subregion features-particularly delta volume/texture-were the strongest contributors to recurrence risk. Together with the transcriptomic enrichment of immunosuppressive programs, these findings indicate that greater post-NAC MPS burden aligns with a hypoxia-associated, immune-evasive phenotype. Only a small subset of immune signatures remained significant after BH correction (q < 0.05), chiefly CD8⁺ T cells and B cells.

In summary, these results link spatial heterogeneity to pathways implicated in treatment resistance, and temporal heterogeneity to immune evasion and pro-recurrence signaling. The concordance between subregion-aware radiomics attribution and transcriptomic programs supports the biological validity and clinical relevance of the proposed imaging signatures.

## Discussion

In this multicenter study of luminal breast cancer, we developed and validated a subregion-aware, multitemporal radiomics model using multiparametric MRI. The models accurately predicted both NAC response and long-term prognosis, outperforming conventional MRI and clinicopathology. The combined models achieved the best performance, with the response classifier showing robust pCR discrimination (AUC 0.92–0.83) and the Cox-XGBoost prognostic model delivering strong prognostic accuracy (C-index up to 0.95). Response prediction was mainly driven by high-perfusion subregional heterogeneity, while temporal changes in moderate-perfusion regions were the strongest prognostic indicators.

The favorable performance of our models likely stems from several methodological features. Built upon both DCE- and DWI-derived multiparametric MRI, our subregion-aware, multitemporal design captured complementary spatial heterogeneity at baseline and temporal changes during NAC. In addition, prior studies have shown that conventional MRI features such as breast edema are associated with poor outcomes in luminal patients receiving NAC. We extended this line of work by refining edema into four categories and evaluating its dynamic changes during NAC, which provided incremental predictive value for both treatment response and prognosis. As a result, combined models integrating radiomics, conventional MRI features, and clinicopathologic factors consistently outperformed single-modality approaches in predicting both NAC response and long-term prognosis.

Several studies have examined longitudinal or multimodal approaches for NAC outcome prediction. Wu and Shi [6, 7] showed that pretreatment DCE-MRI heterogeneity related to response or recurrence, but analyses were limited to single timepoints. More recently, Huang, Gao, and Tang [8, 14] developed deep learning–based spatiotemporal models with high accuracy in multicenter cohorts, yet these subtype-mixed approaches rely on opaque features with limited biological interpretability. As HR-positive and HR-negative tumors differ substantially in chemosensitivity and recurrence biology, their applicability to luminal-specific populations remains uncertain. In contrast, our study focused specifically on luminal breast cancer, validated models across independent cohorts with long-term outcomes, and strengthened interpretability through radiogenomic analyses. By demonstrating that distinct subregional features predominated in response versus prognostic models, we link spatial and temporal imaging heterogeneity to different mechanisms of chemoresistance and recurrence, thereby extending radiogenomic research in luminal breast cancer [15, 16].

The response RadScores were mainly driven by HPS-derived features capturing vascular heterogeneity and asymmetric enhancement, indicative of irregular microvasculature and reduced drug penetration, explaining their association with reduced NAC sensitivity. High-risk groups defined by these scores were enriched for estrogen signaling, PI3K–Akt/mTOR, and cell-cycle/RTK pathways, molecular programs mediating luminal chemoresistance and suggesting potential benefit from targeted agents such as PAM or CDK4/6 inhibitors, as supported by the MONARCH-3 and DAWNA-2 trials [17, 18]. In contrast, prognostic RadScores were dominated by MPS-derived δ-volume and texture changes, reflecting hypoxia-prone habitats reported in prior habitat imaging studies [19, 20]. Transcriptomic analyses confirmed enrichment of TGF-β, angiogenesis, NF-κB, and JAK–STAT pathways [21, 22], aligning with hypoxia-driven immune suppression and recurrence in luminal tumors. Clinically, persistent or expanding MPS burden after NAC may identify patients at elevated recurrence risk who could benefit from intensified surveillance or novel adjuvant strategies targeting hypoxia-associated biology.

From a clinical perspective, our models address two major unmet needs in luminal breast cancer. The response classifier, applied before NAC, can identify patients unlikely to achieve pCR, sparing them ineffective chemotherapy and guiding alternatives such as surgery and endocrine therapy. The prognostic model, applied after NAC, stratifies recurrence risk among non-pCR patients to guide surveillance intensity or adjuvant therapy. Although developed separately, the two models mirror clinical workflows: response prediction informs upfront treatment, while prognostic assessment becomes relevant after therapy. To ensure consistency across varied NAC regimens, prognostic modeling was restricted to non-pCR patients. Together, these tools provide complementary support across treatment stages, and their radiogenomic interpretability suggests value for tailoring trial enrollment and targeted interventions. Nonetheless, prospective validation in larger, more diverse cohorts will be essential before clinical adoption.

Despite its multicenter design with long follow-up, this study has several limitations. First, the retrospective nature may introduce selection bias, and prospective validation is required. Second, although over 500 luminal patients were included, the sample size remains moderate which may affect generalizability. Third, DCE- and DWI-derived subregions were segmented separately; multiparametric co-registration could provide a more integrated assessment of tumor heterogeneity. Fourth, while radiogenomic analyses supported the biological plausibility of our models, experimental validation remains necessary. Finally, although machine learning–based radiomics achieved strong performance, future integration of deep radiomics, pretrained models, and genomic assays may further improve accuracy and facilitate clinical translation.

In summary, our findings establish subregion-aware, multitemporal radiomics as a promising approach for individualized decision-making on treatment response and prognosis in luminal breast cancer.

## Supplementary Information

Below is the link to the electronic supplementary material.

Supplementary Material 1

## Data Availability

Due to patient privacy, individual-level imaging and clinical data cannot be made publicly available but may be obtained from the corresponding author (76218896@qq.com) on reasonable request and with approval from the Institutional Review Board of Fudan University Cancer Center.Radiomic feature extraction complied with IBSI guidelines. The complete feature list, key parameter settings, and model development scripts are available in our public GitHub repository (https://github.com/Oliver950718-ssy/CI).Public datasets used in this study are accessible via:- I-SPY2: 10.7937/TCIA.D8Z0-9T85.

## Abbreviations

ADC: Apparent Diffusion Coefficient
AI: Artificial Intelligence
AUC: Area Under the Curve
BI-RADS: Breast Imaging Reporting and Data System
BPE: Background Parenchymal Enhancement
CI: Confidence Interval
C-index: Concordance Index
CTNeoBC: Collaborative Trials in Neoadjuvant Breast Cancer
DCE-MRI (DCE): Dynamic Contrast-Enhanced Magnetic Resonance Imaging
DFS: Disease-Free Survival
DEG: Differentially Expressed Gene
DWI: Diffusion-Weighted Imaging
ER: Estrogen Receptor
ET: Endocrine Therapy
FC: Fold Change
FDR: False Discovery Rate
FUSCC: Fudan University Shanghai Cancer Center
GSEA: Gene Set Enrichment Analysis
GLCM/GLRLM/GLSZM/GLDM/NGTDM: Texture Matrices used in Radiomics
HER2: Human Epidermal Growth Factor Receptor 2
HR: Hazard Ratio
HPS: High-Perfusion Subregion
ICC: Intraclass Correlation Coefficient
KEGG: Kyoto Encyclopedia of Genes and Genomes
LVI: Lymphovascular Invasion
MPS: Moderate-Perfusion Subregion
MRI: Magnetic Resonance Imaging
NAC: Neoadjuvant Chemotherapy
OR: Odds Ratio
OS: Overall Survival
pCR: Pathological Complete Response
PE: Percentage Enhancement
PR: Progesterone Receptor
RadScore: Radiomic Score
ROC: Receiver Operating Characteristic
RTK: Receptor Tyrosine Kinase
SER: Signal Enhancement Ratio
SHAP: Shapley Additive Explanation
SMOTE: Synthetic Minority Oversampling Technique
ssGSEA: Single-Sample Gene Set Enrichment Analysis
TNBC: Triple-Negative Breast Cancer
VOI/ROI: Volume/Region of Interest
WIS/WOS: Wash-in Slope / Wash-out Slope
