# Supplementary material for: Multicenter study provides radiomic and biological insights into neoadjuvant chemotherapy response and prognosis in luminal breast cancer
Source: Cancer Imaging. 2026 Feb 2;26:34. doi: 10.1186/s40644-026-00994-1 (PMC12951939; doi:10.1186/s40644-026-00994-1)
Supplement: Supplementary file 1 — Supplementary Material 1 [file 40644_2026_994_MOESM1_ESM.docx]

**Supplemental Methods**

# Imaging Protocols

**In the FUSCC cohort** All patients underwent MRI examinations on a 1.5T scanner (Siemens Healthineers, Erlangen, Germany) using a 16-channel phased-array breast surface coil. First, T1-weighted imaging (T1WI), T2-weighted imaging (T2WI), and diffusion-weighted imaging (DWI) (single-shot echo-planar imaging, TR/TE 5600/75 ms; thickness 5.0 mm; b value 0/800 s/mm²) were performed, followed by dynamic contrast-enhanced MRI (DCE-MRI) (spoiled gradient echo, TR/TE 4.4/1.8 ms; thickness 1.5 mm). A contrast agent injected (0.1 mmol/kg, rate of 2.0 ml/s) after a plain scan and was followed immediately by a 20-ml saline flush. Post-contrast images were acquired at 90, 150, 210, 270 and 330 s after injection, at 60-s intervals.

**In the YNCC cohort** All MR examinations were performed on a 1.5 T MRI system (Siemens Healthineers, Erlangen, Germany) using a 16-channel phased-array breast surface coil. Similarly, all patients underwent T1-weighted imaging (T1WI), T2-weighted imaging (T2WI), diffusion-weighted imaging (DWI) (single-shot echo-planar imaging, TR/TE 4800/81 ms; thickness 5.0 mm; b value 0/800 s/mm²), and dynamic contrast-enhanced MRI (DCE-MRI) (spoiled gradient echo, TR/TE 4.4/1.5 ms; thickness 1.5 mm).

**In the I-SPY 2 cohort** The A6698 imaging protocol and the DW-MRI quality assurance process have been previously reported.The MRI component of the ACRIN 6698 trial consisted of four sequential studies: pretreatment (T0), early-treatment (T1), midtreatment (T2), and posttreatment (T3). T2-weighted (T2W), multi-b-value DWI, and DCE acquisitions were taken at each study timepoint. DW-MRI was acquired prior to DCE-MRI in the axial orientation with diffusion gradients in three orthogonal directions using multiple b-values (0, 100, 600, and 800 s/mm2), with a single-shot, diffusion-weighted, spin-echo echo-planar imaging sequence with parallel imaging (reduction factor ≥ 2) and fat suppression. Required scan parameters were TR ≥ 4000 ms, TE minimum (50–100 ms), flip angle 90°, field of view 300–360 mm, acquired matrix 128 × 128 to 192 × 192, and scan time ≤ 5 min. The acquired resolution was 1.7–2.8 mm in-plane with a 4–5 mm slice thickness. No respiratory triggering or other motion compensation methods were used. Test and retest DW-MRI scans for a given patient were performed in the same imaging examination, at either the pre-treatment (preferred) or early treatment timepoint. The patient was positioned normally (prone) and scanned with initial localization, T2W, and DW-MRI acquisitions. They were then removed from the scanner and taken off the scanner bed, then repositioned as before. The full ACRIN 6698 protocol was then performed, consisting of localization, T2W, DW-MRI, and DCE acquisitions.

# Image Preprocessing and Feature Harmonization

To minimize inter-center heterogeneity arising from different scanners and acquisition protocols, all MRI data underwent standardized preprocessing prior to radiomic feature extraction. First, images were resampled to an isotropic voxel size of 1 × 1 × 1 mm³ using B-spline interpolation, which ensures spatial consistency across datasets and facilitates reproducible extraction of three-dimensional features. Resampling reduces variability introduced by anisotropic voxel dimensions and allows shape- and texture-based features to be computed on a comparable spatial grid.

Next, grayscale normalization was performed using Z-score transformation on a per-patient basis, defined as:

$$I_{norm}=\frac{I-\mu}{\sigma}$$

where I is the original voxel intensity, μ is the mean intensity of the image, and σ is the corresponding standard deviation. This step mitigates differences in absolute intensity scales between centers and normalizes the intensity distribution, allowing features derived from first-order statistics and gray-level matrices to remain comparable.

Finally, to further harmonize radiomic features across centers, ComBat harmonization was applied. ComBat is an empirical Bayes framework originally developed for genomic studies and subsequently adapted to radiomics, which adjusts for systematic site/scanner effects while preserving biological variability of interest [1]. The method models feature values as a combination of biological signal, batch effect, and random error, and applies parametric adjustments to remove non-biological variation. In this study, ComBat harmonization was conducted in accordance with the Image Biomarker Standardization Initiative (IBSI) recommendations, thereby reducing center-related bias and improving generalizability of the extracted features across the FUSCC and YNCC cohorts [2].

# Traditional MRI Features Evaluation

The traditional MRI features before and after NAC were evaluated based on T2WI and DCE images according to the Breast Imaging Reporting and Data System (BI-RADS) lexicon and our previous research, including breast edema (no, peritumoral, subcutaneous or prepectoral, and diffuse edema), shrinkage pattern (concentric and non-concentric shrinkage), shape (round or oval, irregular), margins (circumscribed, irregular, spiculated), background parenchymal enhancement (BPE, minimal-mild, moderate, marked), internal enhancement characteristics (homogeneous or rim enhancement, heterogeneous), and multiplicity (unifocal, multifocal, or multicentric). The changes in edema and BPE during NAC were evaluated and recorded as reduced, stable, or increased.

# Region of interest (ROI) Segmentation and Reproducibility Verification

1. **Whole tumor region:** ROIs were placed on all slices of the whole-tumor. For multiple lesions, only the largest lesion was delineated and analysed in the following process. Two radiologists (with 8 and 12 years of experience, respectively) randomly delineated VOIs for 50 patients using 3D Slicer software to validate the repeatability of features. Interobserver repeatability was assessed using the intraclass correlation coefficient (ICC), as follows: poor (0.0–0.50), moderate (0.51–0.75), good (0.76–0.90) and excellent (0.91–1.0) [3].

**(2) Peritumoral region** was defined by expanding 5 mm outward from the tumor's VOI using the "Scipy" package in Python software (version 3.6) [4-5]. Based on the labels of breast tumors, the peritumoral region is defined as the area outside the tumor within n millimeters. In the tumor label, pixels with a value of 1 belong to the tumor region, while pixels with a value of 0 belong to the background region. First, the surface of the breast tumor label is obtained. For 3D data, each pixel has 26 neighboring pixels. Iterate through each pixel in the breast tumor label. If all 26 neighboring pixels of a pixel have a value of 1, it is considered inside the tumor; otherwise, it is considered on the tumor surface. Second, obtain the voxel spacing of the data. The voxel spacing values in the x, y, and z directions are s1, s2, and s3, respectively. Third, calculate the peritumoral region. Iterate through the pixels pi obtained from the first step, assuming the spatial coordinates of this pixel are (xi, yi, zi). Define a large neighborhood search space around this pixel, excluding the tumor region. Iterate through this search space, assuming the coordinates of the pixels inside the search space are (xik, yik, zik). Calculate the Euclidean distance D between each pixel in the search space and pi using the following formula. If D is less than n, the pixel is considered part of the peritumoral region.

$$D=\sqrt{{[s1(xi-xik)]}^{2}+{[s2(yi-xik)]}^{2}+{[s3(zi-zik)]}^{2}}$$

1. **Intratumoral subregions:** For DCE-MRI images, the segmentation method for subregions involves the following steps [6-7]: First, calculate the pixel values of the same pixel within the tumor at different time periods of DCE (pre-, early- and late- contrast injection) based on the time-signal intensity curve (Figure E1). These kinetic features include the wash-in slope (WIS), wash-out slope (WOS), signal enhancement ratio (SER), and percentage enhancement (PE). Then, form a feature vector for each pixel consisting of these four kinetic features. Next, perform clustering on the different feature vectors using unsupervised k-means clustering. The result is that all pixels are divided into three clusters and assigned different colors, representing subregions within the tumor with different kinetic features. Subregion 1 (red) has the lowest values for WIS, WOS, SER, and PE, representing a low perfusion subregion. Subregion 2 (green) has moderate values for WIS, WOS, SER, and PE, representing a moderately perfused subregion. Highly Subregion 3 (blue) has the highest values for WIS, WOS, SER, and PE, representing a highly perfused subregion.

For ADC images, the segmentation method for subregions involves the following steps: First, calculate the pixel values of ADC at each voxel within the tumor. ADC values represent tissue diffusivity and are often used to distinguish different tissue types based on water molecule diffusion. Then, calculate the average ADC value within the region of interest (VOI) in the tumor. Use this average ADC value as the threshold for segmentation. Voxel values greater than the threshold are classified as one subregion, representing areas with lower cellularity or less dense tissue, while those below the threshold are classified as another subregion, indicating regions with higher cellularity or potential necrosis. Afterward, apply morphological operations such as dilation or erosion to refine the subregion boundaries and remove small isolated areas. Finally, extract features such as volume, average ADC, and shape for each segmented subregion.

# Radiomics Features Extraction

A total of 873 multiregion radiomic features were extracted from each ROI using the Pyradiomics package (V3.0) in Python (V3.6) (Table E1) according to the Image Biomarker Standardization Initiative, including 14 shape, 18 first-order features, 75 texture features and 744 wavelet features [11-12]. First-order features describe the distribution of voxel intensities, while texture features are obtained from five texture matrices to describe the gray-level distribution relationships of pixels and their surrounding spatial neighborhoods. Eight wavelet filters are applied to the original images to extract wavelet features of first-order and texture features (LLL, LLH, LHL, HLL, LHH, HLH, HHH) (Table E1).

# Feature Selection and Model Development

For response, we performed an outer 5-fold CV. In each outer split, the inner loop used 5-fold CV repeated 10 times (50 resamples). At each inner resample, an XGBoost (binary:logistic) model was trained on the screened features; gain importance was computed and features were ranked. Candidate subsets were defined as (i) top-N features with N∈{5,7,10,15}, and (ii) minimal prefix whose cumulative gain reached ≥90%. Each candidate subset was refit and evaluated on the corresponding inner validation fold by AUC; the best subset per resample was recorded. Per-feature selection frequency was calculated over all inner resamples. Features with frequency ≥0.60 formed the “stable” subset. The fold-specific final classifier was trained on the whole outer-training fold using only the stable subset and evaluated on the outer-test fold to obtain out-of-fold (OOF) predictions and AUC.

For prognosis, we used the same outer/inner structure with XGBoost objective set to survival:cox. Candidate subsets were evaluated by Harrell’s C-index, the frequency threshold remained 0.60, and the maximum subset size was 15.

# Cutoff selection details

**Response (pCR).** We defined a resistance score as R = 1 − p̂(pCR). The probability cutoff was selected in the training cohort by maximizing the Youden index (sensitivity + specificity − 1). The threshold and its 95% CI were estimated by 1,000 bootstrap resamples and then fixed a priori and applied unchanged to all evaluation cohorts (no re-tuning).

**Prognosis (DFS).** We used the continuous risk score from the Cox-XGBoost model. The split point was selected in the training cohort by maximizing the time-dependent Youden index at 5 years (based on the time-dependent ROC). The threshold and its 95% CI were obtained via 1,000 bootstrap resamples and then applied unchanged to external cohorts for stratification.

# References

1. Leithner D, Nevin RB, Gibbs P et al. ComBat Harmonization for MRI Radiomics: Impact on Nonbinary Tissue Classification by Machine Learning. Invest Radiol. 2023 Sep 1;58(9):697-701.
2. Zwanenburg A, Vallières M, Abdalah MA et al. The Image Biomarker Standardization Initiative: Standardized Quantitative Radiomics for High-Throughput Image-based Phenotyping. Radiology. 2020 May;295(2):328-338.
3. Koo TK, Li MY. A Guideline of Selecting and Reporting Intraclass Correlation Coefficients for Reliability Research. J Chiropr Med. 2016 Jun;15(2):155-63.
4. Su GH, Xiao Y, Jiang L et al. Radiomics features for assessing tumor-infiltrating lymphocytes correlate with molecular traits of triple-negative breast cancer. J Transl Med. 2022 Oct 15;20(1):471.
5. Wang S, Sun Y, Li R et al. Diagnostic performance of perilesional radiomics analysis of contrast-enhanced mammography for the differentiation of benign and malignant breast lesions. Eur Radiol. 2022 Jan;32(1):639-649.
6. Wu J, Gong G, Cui Y et al. Intratumor partitioning and texture analysis of dynamic contrast-enhanced (DCE)-MRI identifies relevant tumor subregions to predict pathological response of breast cancer to neoadjuvant chemotherapy. J Magn Reson Imaging. 2016 Nov;44(5):1107-111.

Wu J, Cao G, Sun X et al. Intratumoral Spatial Heterogeneity at Perfusion MR Imaging Predicts Recurrence-free Survival in Locally Advanced Breast Cancer Treated with Neoadjuvant Chemotherapy. Radiology. 2018 Jul;288(1):26-35.

# Figures


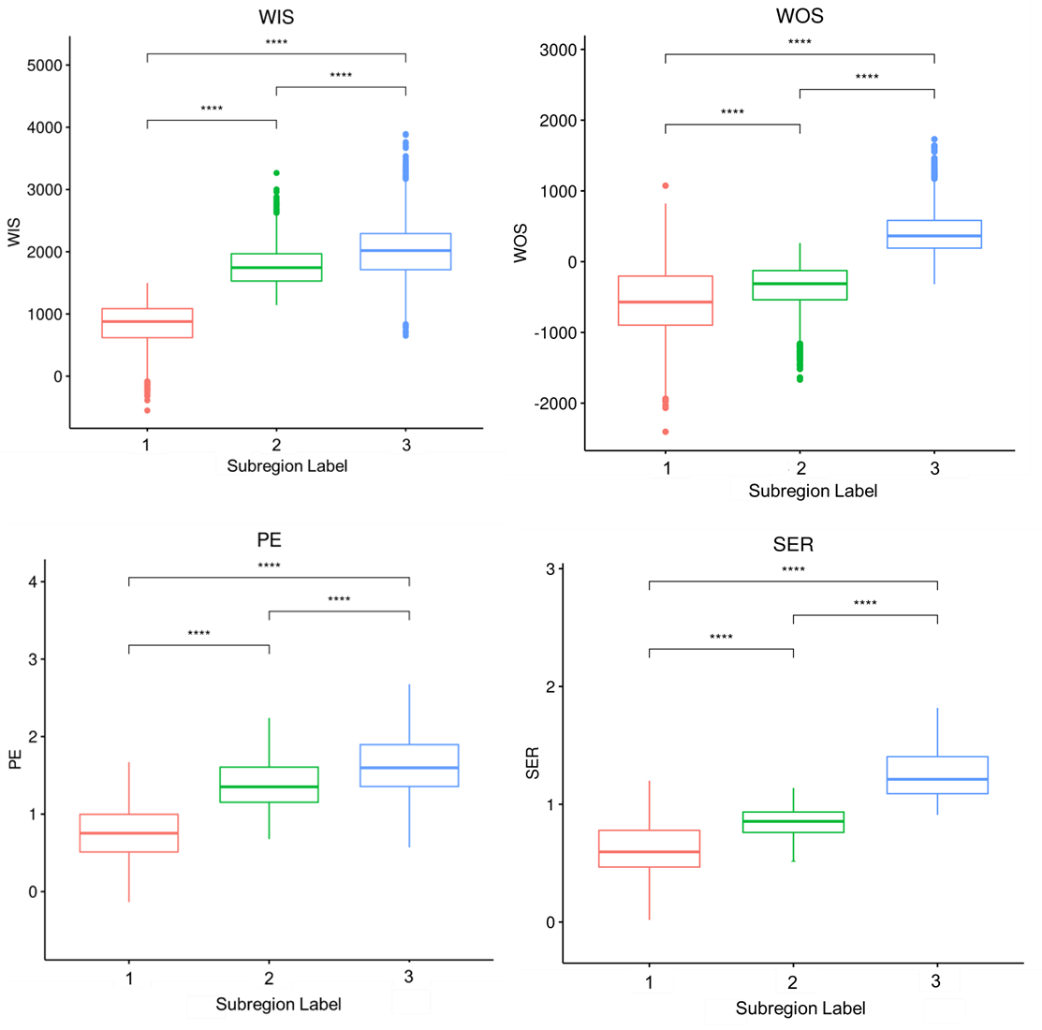


**Figure E1: Box plots show distribution of four perfusion imaging parameters for three DCE intratumoral subregions.**

The pixel values at the same location within the tumor were extracted to form feature vectors, and four dynamic characteristics for each pixel were calculated: wash-in slope (WIS), wash-out slope (WOS), signal enhancement ratio (SER), and percentage enhancement (PE). These feature vectors underwent unsupervised k-means clustering analysis. Three clusters (i.e., subregions) better reflected the tumor's microvasculature distribution: poorly perfused (minimum values of WIS, WOS, SER, PE), highly perfused (maximum values), and moderately perfused (intermediate values).


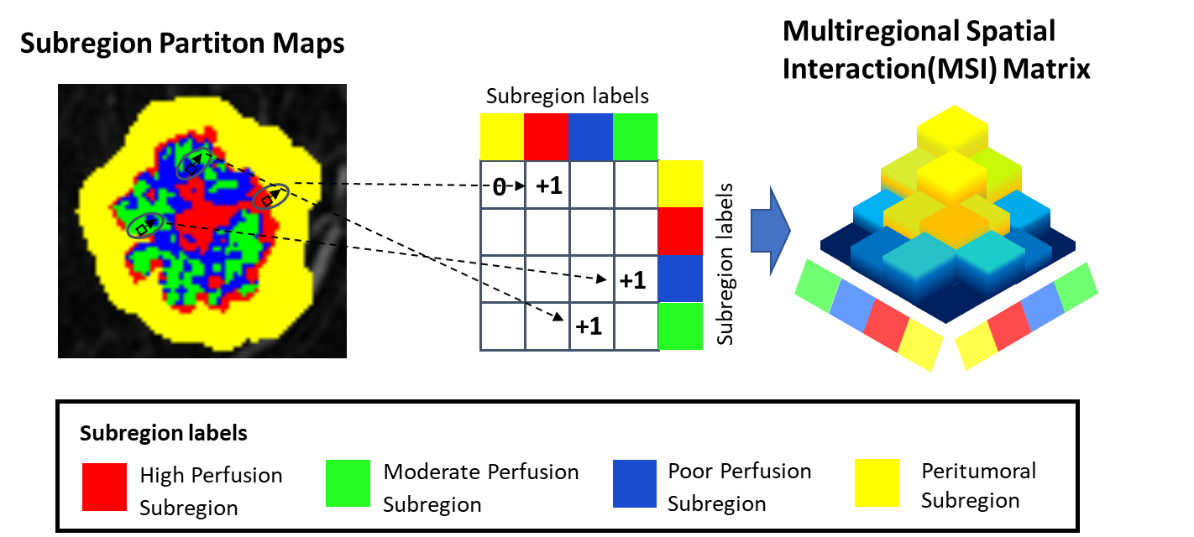


**Figure E2: Diagram illustrating multiregional spatial interaction (MSI) feature extraction**

(a) Numerical labels "0, 1, 2, 3" were assigned to differentiate different types of voxels within the three subregions and the peritumoral region. Then, the position and number of each voxel were counted in three-dimensional space concerning neighboring voxels and recorded these counts in the corresponding positions of a 4×4 matrix. (b) This process was repeated iteratively for all voxels within the tumor, eventually generating the MSI matrix to comprehensively represent the degree and extent of tumor spatial heterogeneity. Subsequently, 22 radiomic features were extracted from the MSI matrix.


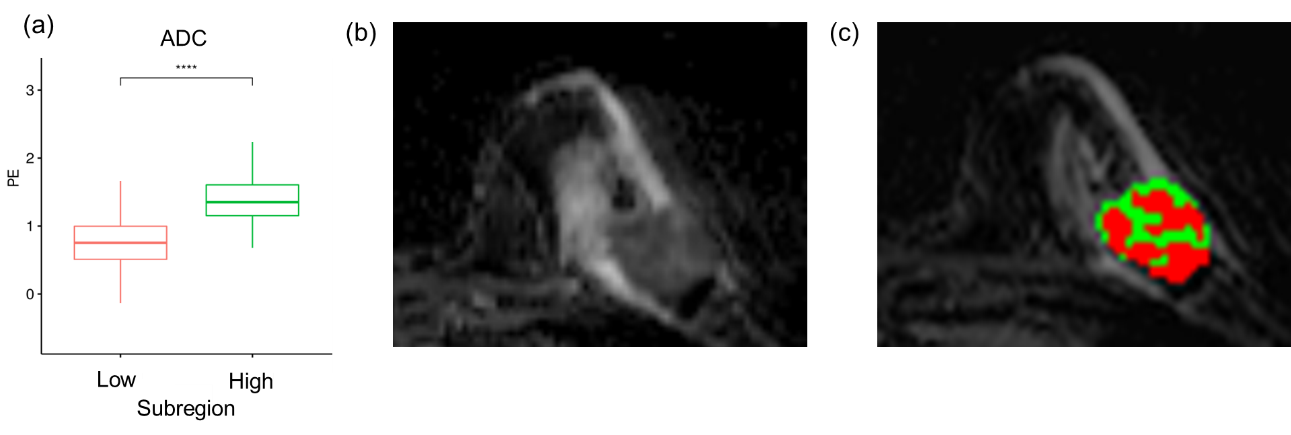


**Figure E3: Box plots show distribution of ADC value of intratumoral subregions.**

(a) Mean values of VOI endosomes were used as thresholds to partition the subregions. There is a difference in ADC values for low cellularity subregion and high cellularity subregion. (b) ADC image before subregion segmentation, (c) ADC image after subregion segmentation, red color represents low cellularity subregion while green color represents high cellularity subregion.


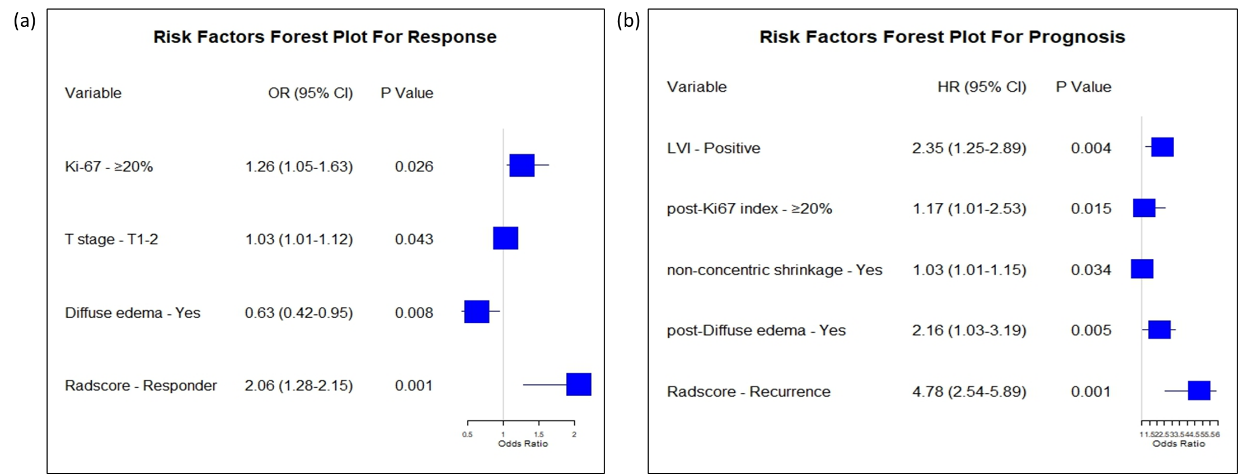


**Figure E4.** Forest plots of independent risk factors for NAC response and prognosis.

(a) Multivariable logistic regression for NAC response showed that Ki-67, T stage, and diffuse edema were associated with treatment sensitivity, while RadScore remained an independent predictor with the strongest effect. (b) Multivariable Cox regression for prognosis identified lymphovascular invasion (LVI), post-NAC Ki-67, non-concentric shrinkage, and post-NAC diffuse edema as risk factors, with RadScore emerging as the most significant independent predictor of recurrence.


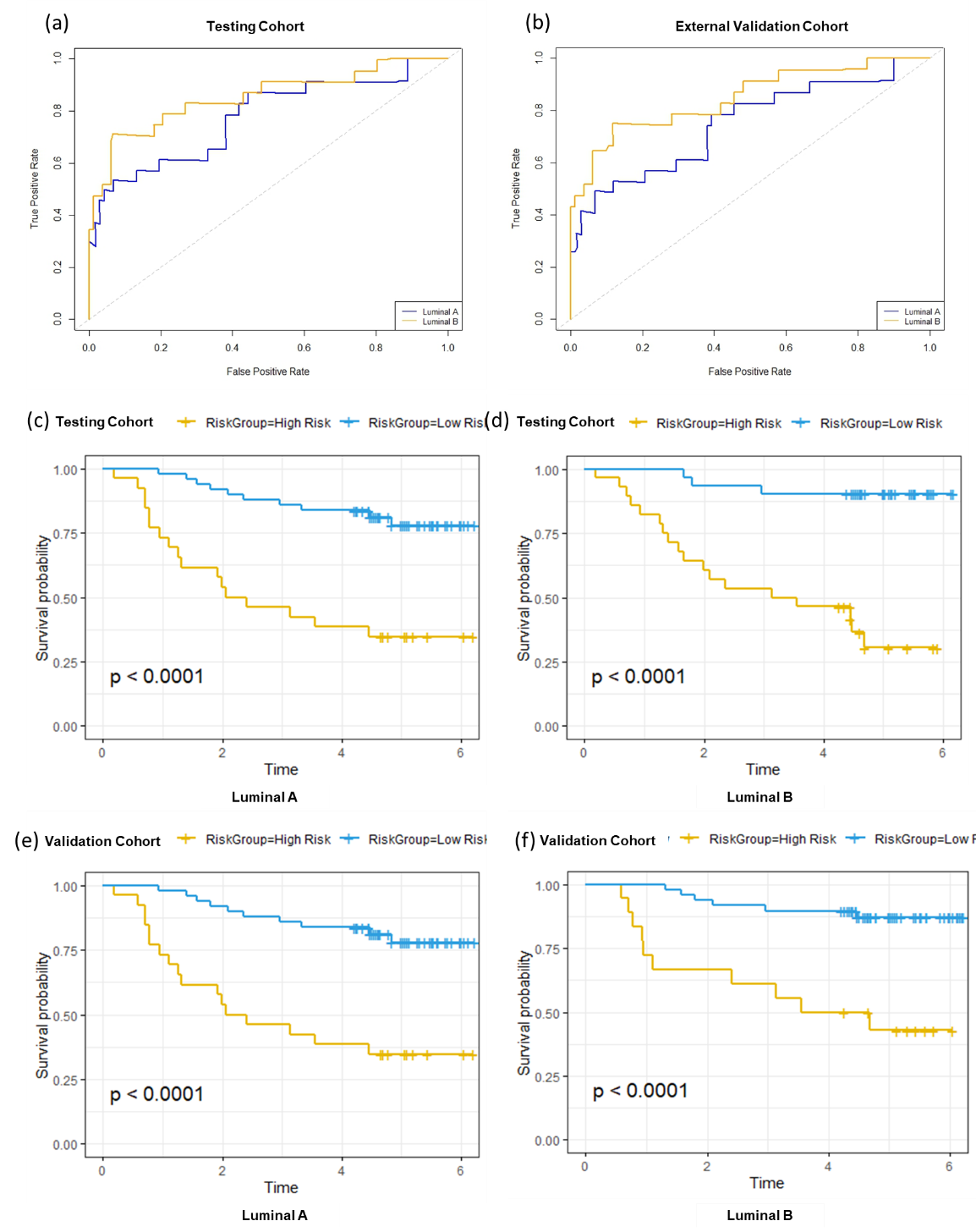
\

**Figure E5.** Subgroup analyses by luminal subtype.

(a–b) ROC curves for NAC response prediction in Luminal A and Luminal B patients in the testing cohort (a) and external validation cohort (b). (c–f) Kaplan–Meier survival curves showing significant separation of high- and low-risk groups stratified by RadScore in Luminal A (c, e) and Luminal B (d, f) patients from the testing and validation cohorts.

**
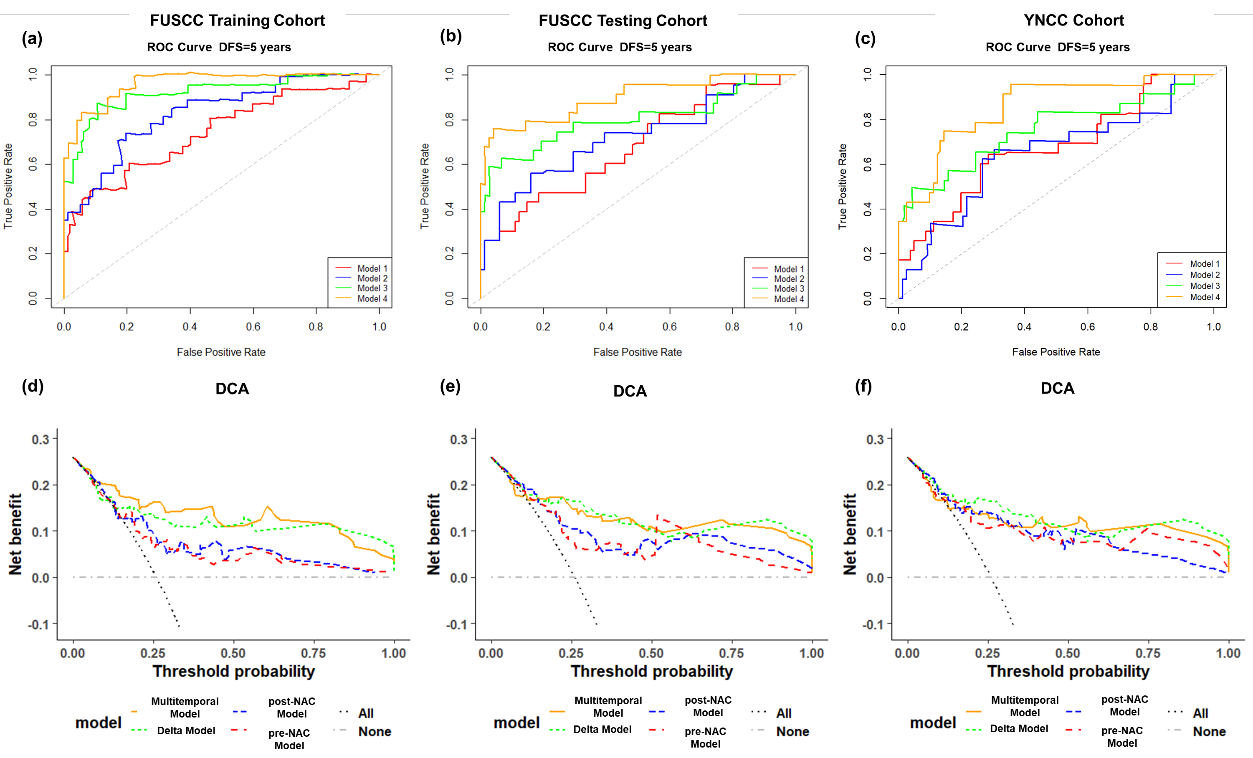
**

**Figure E6.** Comparison of prognostic models based on different temporal features.

(a–c) ROC curves for 5-year DFS prediction in the FUSCC training (a), FUSCC testing (b), and YNCC validation (c) cohorts. Models included pre-NAC, post-NAC, Δ (post–pre), and multitemporal feature sets. (d–f) Decision curve analyses (DCA) demonstrate that the multitemporal model provided the highest net clinical benefit across all cohorts.

**Supplemental Tables**

| **Table E1**. The explanation of radiomic features in this study. | |
| --- | --- |
| Feature types | Radiomics Significance |
| Original_Shape | original_shape_Elongation |
| (n=14) | original_shape_Flatness |
|  | original_shape_LeastAxisLength |
|  | original_shape_MajorAxisLength |
|  | original_shape_Maximum2DDiameterColumn |
|  | original_shape_Maximum2DDiameterRow |
|  | original_shape_Maximum2DDiameterSlice |
|  | original_shape_Maximum3DDiameter |
|  | original_shape_MeshVolume |
|  | original_shape_MinorAxisLength |
|  | original_shape_Sphericity |
|  | original_shape_SurfaceArea |
|  | original_shape_SurfaceVolumeRatio |
|  | original_shape_VoxelVolume |
| First-Order | original_firstorder_10Percentile |
| (n=18) | original_firstorder_90Percentile |
|  | original_firstorder_Energy |
|  | original_firstorder_Entropy |
|  | original_firstorder_InterquartileRange |
|  | original_firstorder_Kurtosis |
|  | original_firstorder_Maximum |
|  | original_firstorder_MeanAbsoluteDeviation |
|  | original_firstorder_Mean |
|  | original_firstorder_Median |
|  | original_firstorder_Minimum |
|  | original_firstorder_Range |
|  | original_firstorder_RobustMeanAbsoluteDeviation |
|  | original_firstorder_RootMeanSquared |
|  | original_firstorder_Skewness |
|  | original_firstorder_TotalEnergy |
|  | original_firstorder_Uniformity |
|  | original_firstorder_Variance |
| GLCM | lbp-2D_glcm_Autocorrelation |
| (n=24) | lbp-2D_glcm_ClusterProminence |
|  | lbp-2D_glcm_ClusterShade |
|  | lbp-2D_glcm_ClusterTendency |
|  | lbp-2D_glcm_Contrast |
|  | lbp-2D_glcm_Correlation |
|  | lbp-2D_glcm_DifferenceAverage |
|  | lbp-2D_glcm_DifferenceEntropy |
|  | lbp-2D_glcm_DifferenceVariance |
|  | lbp-2D_glcm_Id |
|  | lbp-2D_glcm_Idm |
|  | lbp-2D_glcm_Idmn |
|  | lbp-2D_glcm_Idn |
|  | lbp-2D_glcm_Imc1 |
|  | lbp-2D_glcm_Imc2 |
|  | lbp-2D_glcm_InverseVariance |
|  | lbp-2D_glcm_JointAverage |
|  | lbp-2D_glcm_JointEnergy |
|  | lbp-2D_glcm_JointEntropy |
|  | lbp-2D_glcm_MCC |
|  | lbp-2D_glcm_MaximumProbability |
|  | lbp-2D_glcm_SumAverage |
|  | lbp-2D_glcm_SumEntropy |
|  | lbp-2D_glcm_SumSquares |
| GLDM | lbp-2D_gldm_DependenceEntropy |
| (n=14) | lbp-2D_gldm_DependenceNonUniformity |
|  | lbp-2D_gldm_DependenceNonUniformityNormalized |
|  | lbp-2D_gldm_DependenceVariance |
|  | lbp-2D_gldm_GrayLevelNonUniformity |
|  | lbp-2D_gldm_GrayLevelVariance |
|  | lbp-2D_gldm_HighGrayLevelEmphasis |
|  | lbp-2D_gldm_LargeDependenceEmphasis |
|  | lbp-2D_gldm_LargeDependenceHighGrayLevelEmphasis |
|  | lbp-2D_gldm_LargeDependenceLowGrayLevelEmphasis |
|  | lbp-2D_gldm_LowGrayLevelEmphasis |
|  | lbp-2D_gldm_SmallDependenceEmphasis |
|  | lbp-2D_gldm_SmallDependenceHighGrayLevelEmphasis |
|  | lbp-2D_gldm_SmallDependenceLowGrayLevelEmphasis |
| GLRLM | lbp-2D_glrlm_GrayLevelNonUniformity |
| (n=16) | lbp-2D_glrlm_GrayLevelNonUniformityNormalized |
|  | lbp-2D_glrlm_GrayLevelVariance |
|  | lbp-2D_glrlm_HighGrayLevelRunEmphasis |
|  | lbp-2D_glrlm_LongRunEmphasis |
|  | lbp-2D_glrlm_LongRunHighGrayLevelEmphasis |
|  | lbp-2D_glrlm_LongRunLowGrayLevelEmphasis |
|  | lbp-2D_glrlm_LowGrayLevelRunEmphasis |
|  | lbp-2D_glrlm_RunEntropy |
|  | lbp-2D_glrlm_RunLengthNonUniformity |
|  | lbp-2D_glrlm_RunLengthNonUniformityNormalized |
|  | lbp-2D_glrlm_RunPercentage |
|  | lbp-2D_glrlm_RunVariance |
|  | lbp-2D_glrlm_ShortRunEmphasis |
|  | lbp-2D_glrlm_ShortRunHighGrayLevelEmphasis |
|  | lbp-2D_glrlm_ShortRunLowGrayLevelEmphasis |
| GLSZM | lbp-2D_glszm_GrayLevelNonUniformity |
| (n=16) | lbp-2D_glszm_GrayLevelNonUniformityNormalized |
|  | lbp-2D_glszm_GrayLevelVariance |
|  | lbp-2D_glszm_HighGrayLevelZoneEmphasis |
|  | lbp-2D_glszm_LargeAreaEmphasis |
|  | lbp-2D_glszm_LargeAreaHighGrayLevelEmphasis |
|  | lbp-2D_glszm_LargeAreaLowGrayLevelEmphasis |
|  | lbp-2D_glszm_LowGrayLevelZoneEmphasis |
|  | lbp-2D_glszm_SizeZoneNonUniformity |
|  | lbp-2D_glszm_SizeZoneNonUniformityNormalized |
|  | lbp-2D_glszm_SmallAreaEmphasis |
|  | lbp-2D_glszm_SmallAreaHighGrayLevelEmphasis |
|  | lbp-2D_glszm_SmallAreaLowGrayLevelEmphasis |
|  | lbp-2D_glszm_ZoneEntropy |
|  | lbp-2D_glszm_ZonePercentage |
|  | lbp-2D_glszm_ZoneVariance |
| NGTDM | lbp-2D_ngtdm_Busyness |
| (n=5) | lbp-2D_ngtdm_Coarseness |
|  | lbp-2D_ngtdm_Complexity |
|  | lbp-2D_ngtdm_Contrast |
|  | lbp-2D_ngtdm_Strength |

| **Table E2**. Inter-reader agreement for traditional MRI features | | | | |
| --- | --- | --- | --- | --- |
| Feature | Scale / Categories | Agreement metric | κ (95% CI) | Percent agreement |
| BPE (pre-NAC) | Minimal, Mild, Moderate, Marked | Weighted κ | 0.79 (0.73–0.85) | 87% |
| Edema pattern (pre-NAC) | None, Peritumoral, Subcutaneous, Prepectoral, Diffuse | Cohen’s κ | 0.76 (0.69–0.83) | 85% |
| Internal enhancement pattern | Homogeneous, Heterogeneous, Clustered ring | Cohen’s κ | 0.73 (0.66–0.80) | 88% |
| Tumor margins | Circumscribed, Non-circumscribed/Spiculated | Cohen’s κ | 0.81 (0.75–0.87) | 90% |
| Tumor shape | Round/Oval, Irregular | Cohen’s κ | 0.83 (0.77–0.89) | 92% |
| Edema (post-NAC) | None, Peritumoral, Subcutaneous, Prepectoral, Diffuse | Cohen’s κ | 0.85 (0.79–0.91) | 93% |
| Shrinkage pattern (post-NAC) | Concentric, Non-concentric shrinkage | Cohen’s κ | 0.78 (0.71–0.85) | 89% |
| Δ BPE during NAC | Reduced, Stable, Increased | Weighted κ | 0.75 (0.68–0.82) | 84% |
| Δ Edema during NAC | Reduced, Stable, Increased | Weighted κ | 0.72 (0.65–0.79) | 82% |
| Agreement between two radiologists for qualitative MRI descriptors used in this study. Agreement is summarized using Cohen’s κ for nominal variables and weighted κ for ordinal variables. All features show good to excellent agreement. Three-line table; no shading.  Interpretation: κ 0.61–0.80 = substantial agreement; κ > 0.80 = almost perfect agreement (Landis & Koch). | | | | |

| **Table E3**. Response-related RadScore | | | | | |
| --- | --- | --- | --- | --- | --- |
| Canonical code | Feature label | Subregion | Modality | Timepoint | Weight |
| pre_dce_hps_ClusterShade | Cluster Shade | High-perfusion | DCE (*) | Pre (+) | 0.852 |
| pre_dce_hps_LAHGLE | LAHGLE | High-perfusion | DCE (*) | Pre (+) | 0.782 |
| pre_adc_highcell_MaximumProbability | Maximum Probability | High-cellularity | ADC (#) | Pre (+) | 0.726 |
| pre_dce_hps_Entropy | Entropy | High-perfusion | DCE (*) | Pre (+) | 0.600 |
| pre_dce_peritumor_Contrast | Contrast | Peritumoral | DCE (*) | Pre (+) | 0.556 |
| pre_adc_wholeRegion_DifferenceEntropy | Difference Entropy | Whole region | ADC (#) | Pre (+) | 0.527 |
| pre_dce_wholeTumor_ZonePercentage | Zone Percentage | Whole tumor | DCE (*) | Pre (+) | 0.194 |
| DCE-derived; #ADC-derived; +Pre-NAC. LAHGLE: Large Area High Gray Level Emphasis. | | | | | |

| **Table E4.** Univariable and multivariable logistic regression analyses of traditional MRI and clinical variables for pCR | | | | |
| --- | --- | --- | --- | --- |
| Variable | Univariable OR (95% CI) | *P*-value | Multivariable OR (95% CI) | *P*-value |
| Age (per 10 years) | 0.95 (0.80–1.12) | 0.55 | 0.97 (0.82–1.16) | 0.74 |
| Tumor size (per cm) | **0.88 (0.78–0.99)** | **0.037** | 0.91 (0.80–1.05) | 0.20 |
| T stage (T1–2 vs T3–4) | **0.85 (0.73–0.98)** | **0.028** | **0.93 (0.46–0.96)** | **0.003** |
| N stage (N0–1 vs N2–3) | 0.82 (0.55–1.23) | 0.34 | 0.90 (0.57–1.41) | 0.63 |
| Histologic grade (G3 vs G1–2) | 1.25 (0.85–1.85) | 0.25 | 1.12 (0.71–1.78) | 0.62 |
| ER expression (per 10%) | 0.96 (0.90–1.02) | 0.17 | 0.98 (0.92–1.05) | 0.61 |
| PR expression (per 10%) | 0.95 (0.90–1.01) | 0.09 | 0.98 (0.92–1.04) | 0.44 |
| Ki-67 index (per 10%) | **1.28 (1.12–1.47)** | **0.001** | **1.34 (1.16–1.71)** | **0.005** |
| LVI (present vs absent) | 0.92 (0.66–1.27) | 0.60 | 0.98 (0.68–1.41) | 0.92 |
| BPE (mod/marked vs min/mild) | 0.88 (0.66–1.16) | 0.36 | 0.95 (0.68–1.33) | 0.76 |
| Tumor shape (irregular vs round/oval) | 0.84 (0.60–1.18) | 0.31 | 0.92 (0.63–1.36) | 0.68 |
| Margins (spiculated vs others) | 0.78 (0.55–1.11) | 0.17 | 0.90 (0.60–1.35) | 0.61 |
| Internal enhancement (heterogeneous/CR vs others) | 0.75 (0.53–1.05) | 0.095 | 0.86 (0.58–1.28) | 0.45 |
| Pre-NAC edema: Peritumoral (vs none) | 0.90 (0.70–1.17) | 0.44 | 0.92 (0.68–1.25) | 0.59 |
| Pre-NAC edema: Subcutaneous (vs none) | 0.85 (0.64–1.13) | 0.25 | 0.88 (0.64–1.22) | 0.45 |
| Pre-NAC edema: Subcutaneous or prepectoral (vs none) | 0.78 (0.58–1.06) | 0.08 | 0.79 (0.58–1.08) | 0.14 |
| Pre-NAC edema: Diffuse (vs none) | **0.62 (0.41–0.92)** | **0.018** | **0.86 (0.33–0.93)** | **0.007** |
| **Selection:** variables with univariable p < 0.10 entered the multivariable model.  **Abbreviations:** OR, odds ratio; CI, confidence interval; LVI, lymphovascular invasion; BPE, background parenchymal enhancement; CR, clustered ring. For continuous variables, ORs are per 10 percentage-point increase unless otherwise specified. | | | | |

| **Table E5.** Prognosis-related RadScore | | | | | |
| --- | --- | --- | --- | --- | --- |
| Canonical code | Feature label | Subregion | Modality | Timepoint | Weight |
| delta_dce_mps_MeshVolume | Mesh Volume | Moderate-perfusion | DCE (*) | Delta (Δ) | 1.260 |
| post_dce_hps_ClusterShade | Cluster Shade | High-perfusion | DCE (*) | Post (++) | 0.795 |
| delta_adc_highcell_Mean | Mean | High-cellularity | ADC (#) | Delta (Δ) | 0.598 |
| delta_dce_mps_JointEntropy | Joint Entropy | Moderate-perfusion | DCE (*) | Delta (Δ) | 0.595 |
| post_dce_mps_MeshVolume | Mesh Volume | Moderate-perfusion | DCE (*) | Post (++) | 0.424 |
| delta_dce_peritumor_Contrast | Contrast | Peritumoral | DCE (*) | Delta (Δ) | 0.399 |
| post_adc_wholeTumor_ZoneEntropy | Zone Entropy | Whole tumor | ADC (#) | Post (++) | 0.386 |
| delta_adc_highcell_GLNN | GLNN | High-cellularity | ADC (#) | Delta (Δ) | 0.274 |
| pre_dce_mps_HGLRE | HGLRE | Moderate-perfusion | DCE (*) | Pre (+) | 0.163 |
| DCE-derived; #ADC-derived; +Pre-NAC; ++Post-NAC; Δ = Delta features. GLNN: Gray Level Non-Uniformity Normalized. HGLRE: High Gray Level Run Emphasis. | | | | | |

| **Table E6.** Univariable and multivariable Cox regression analyses of traditional MRI and clinical variables for pCR | | | | |
| --- | --- | --- | --- | --- |
| Variable | Univariable HR (95% CI) | *P*-value | Multivariable HR (95% CI) | *P*-value |
| Age (per 10 years) | 1.08 (0.95–1.22) | 0.24 | 1.04 (0.91–1.20) | 0.55 |
| Tumor size, pre-NAC (per cm) | 1.16 (1.05–1.28) | 0.004 | 1.08 (0.97–1.21) | 0.16 |
| T stage (per stage) | 1.32 (1.11–1.57) | 0.001 | 1.18 (0.99–1.42) | 0.064 |
| N stage (node-positive vs negative) | 1.48 (1.10–2.00) | 0.010 | 1.22 (0.89–1.67) | 0.21 |
| Histologic grade (G3 vs G1–2) | 1.28 (0.95–1.72) | 0.10 | 1.10 (0.81–1.49) | 0.54 |
| ER expression (per 10%) | 0.96 (0.90–1.02) | 0.19 | 0.98 (0.92–1.05) | 0.61 |
| PR expression (per 10%) | 0.95 (0.90–1.01) | 0.09 | 0.99 (0.93–1.06) | 0.84 |
| Post-NAC Ki-67 index (per 10%) | **2.21 (1.31–3.72)** | **0.003** | **3.13 (1.38–7.10)** | **0.007** |
| LVI (present vs absent) | **1.90 (1.11–3.24)** | **0.018** | **1.78 (1.04–4.21)** | **0.011** |
| Pre-NAC BPE (mod/marked vs min/mild) | 1.26 (1.01–1.57) | 0.040 | 1.10 (0.86–1.41) | 0.44 |
| Pre-NAC edema: peritumoral (vs none) | 1.18 (0.92–1.51) | 0.19 | 1.06 (0.81–1.39) | 0.66 |
| Pre-NAC edema: subcutaneous (vs none) | 1.24 (0.96–1.61) | 0.10 | 1.08 (0.81–1.43) | 0.61 |
| Pre-NAC edema: subcutaneous or prepectoral (vs none) | 1.31 (1.00–1.72) | 0.049 | 1.12 (0.84–1.49) | 0.45 |
| Pre-NAC edema: diffuse (vs none) | 1.42 (1.08–1.87) | 0.012 | 1.16 (0.88–1.54) | 0.30 |
| Post-NAC BPE (mod/marked vs min/mild) | 1.34 (0.36–1.69) | 1.14 | 1.12 (0.88–1.43) | 0.36 |
| Post-NAC edema: subcutaneous or prepectoral (vs none) | **1.28 (1.13–1.45)** | **<0.001** | **1.16 (1.02–1.33)** | **0.031** |
| Post-NAC diffuse edema (present vs absent) | **4.92 (2.10–11.40)** | **<0.001** | **6.42 (2.33–7.66)** | **<0.001** |
| Shrinkage pattern (non-concentric vs concentric) | **1.90 (1.10–3.30)** | **0.019** | **2.85 (1.15–5.07)** | **0.024** |
| Δ BPE: reduced (vs stable) | 0.78 (0.63–0.98) | 0.034 | 0.86 (0.69–1.08) | 0.19 |
| Δ BPE: increased (vs stable) | 1.41 (0.49–1.82) | 1.39 | 1.18 (0.91–1.54) | 0.21 |
| Δ edema: reduced (vs stable) | 0.76 (0.60–0.96) | 0.022 | 0.88 (0.68–1.13) | 0.31 |
| Δ edema: increased (vs stable) | 1.38 (1.06–1.79) | 0.016 | 1.19 (0.91–1.56) | 0.20 |
| **Selection:** variables with univariable p < 0.10 entered the multivariable Cox model. BPE and edema changes during NAC were categorized as reduced, stable (reference), or increased.  **Abbreviations:** HR, hazard ratio; CI, confidence interval; LVI, lymphovascular invasion; BPE, background parenchymal enhancement; Δ, change during NAC; DFS, disease-free survival. Continuous variables: age per 10 years, ER/PR/Ki-67 per 10 percentage points, tumor size per cm. | | | | |

| Table E7. Cutoff of models for response and recurrence | | | | | | |
| --- | --- | --- | --- | --- | --- | --- |
| Endpoint | Score | Selection rule | Cutoff  (95% CI) | Internal training set: Sens | Internal training set: Spec | Internal training set: AUC/C-index (95% CI) |
| pCR (binary) | RadScore_resp (non-pCR probability = 1 − p̂(pCR)) | Youden (training) | τ_resp = 0.62 (0.58–0.66) (equiv. p̂(pCR) cutoff ≈ 0.38)* | 0.80 (0.72–0.86) | 0.79 (0.73–0.84) | AUC = 0.90 (0.85–0.94) |
| DFS (time-to-event) | RadScore_prog (prognostic risk score from Cox-XGBoost; higher = higher recurrence hazard) | 5-year td-Youden (training) | τ_prog = 0.41 (0.37–0.46)# | 0.76 (0.66–0.85) | 0.81 (0.74–0.86) | C-index = 0.92 (0.83–0.97) |
| External predictive and prognostic performance assessed in YNCC; I-SPY2 used for biological analysis only.  *Patients were labeled high non-pCR risk if RadScore_resp ≥ 0.62 (equivalently, p̂(pCR) ≤ 0.38).  #Patients were labeled high recurrence risk if RadScore_prog ≥ 0.41; note that RadScore_prog is a risk score (hazard-oriented), not a probability. | | | | | | |

| **Table E8.** Response-related enrichment pathway | | | | | | |  | |  |
| --- | --- | --- | --- | --- | --- | --- | --- | --- | --- |
| No. | Pathway | Direction | −log10(FDR) | Set size | P value | FDR (q) | |  |  |
| 1 | Drug metabolism | Up | 2.52 | 86 | 0.002 | 0.003 | |  |  |
| 2 | PI3K–Akt signaling pathway | Up | 2.10 | 132 | 0.005 | 0.008 | |  |  |
| 3 | Estrogen signaling pathway | Up | 1.96 | 96 | 0.008 | 0.011 | |  |  |
| 4 | ABC transporters | Up | 1.85 | 55 | 0.010 | 0.014 | |  |  |
| 5 | Hypoxia pathway | Up | 1.72 | 78 | 0.013 | 0.019 | |  |  |
| 6 | MAPK signaling pathway | Up | 1.60 | 128 | 0.017 | 0.025 | |  |  |
| 7 | EMT program | Up | 1.55 | 64 | 0.020 | 0.028 | |  |  |
| 8 | Notch signaling pathway | Up | 1.47 | 72 | 0.024 | 0.034 | |  |  |
| 9 | DNA repair | Up | 1.43 | 60 | 0.026 | 0.037 | |  |  |
| 10 | Glycolysis / gluconeogenesis | Up | 1.37 | 66 | 0.030 | 0.043 | |  |  |
| 11 | mTOR signaling | Up | 1.31 | 85 | 0.034 | 0.049 | |  |  |
| 12 | Oxidative phosphorylation | Down | 1.74 | 102 | 0.013 | 0.018 | |  |  |
| 13 | cGMP–PKG signaling | Down | 1.70 | 120 | 0.014 | 0.020 | |  |  |
| 14 | Apoptosis | Down | 1.59 | 88 | 0.018 | 0.026 | |  |  |
| 15 | p53 signaling pathway | Down | 1.54 | 73 | 0.020 | 0.029 | |  |  |
| 16 | cAMP signaling pathway | Down | 1.51 | 76 | 0.022 | 0.031 | |  |  |
| 17 | Fatty acid metabolism | Down | 1.40 | 65 | 0.028 | 0.040 | |  |  |
| 18 | Focal adhesion | Down | 1.39 | 108 | 0.029 | 0.041 | |  |  |
| 19 | Peroxisome | Down | 1.35 | 59 | 0.032 | 0.045 | |  |  |
| 20 | Ubiquitin-mediated proteolysis | Down | 1.33 | 91 | 0.033 | 0.047 | |  |  |

| **Table E9.** Recurrence -related enrichment pathway | | | | | | |
| --- | --- | --- | --- | --- | --- | --- |
| No. | Pathway | Direction | −log10(FDR) | Set size | P value | FDR (q) |
| 1 | Cytokine–chemokine signaling | Up | 2.40 | 150 | 0.002 | 0.004 |
| 2 | JAK–STAT signaling pathway | Up | 2.05 | 110 | 0.005 | 0.009 |
| 3 | NF-κB signaling pathway | Up | 1.92 | 120 | 0.008 | 0.012 |
| 4 | Angiogenesis | Up | 1.82 | 92 | 0.010 | 0.015 |
| 5 | TGF-β signaling | Up | 1.66 | 84 | 0.015 | 0.022 |
| 6 | PPAR signaling | Up | 1.52 | 74 | 0.021 | 0.030 |
| 7 | Complement cascade | Up | 1.40 | 52 | 0.028 | 0.040 |
| 8 | T-cell receptor signaling pathway | Down | 2.30 | 105 | 0.003 | 0.005 |
| 9 | B-cell receptor signaling pathway | Down | 1.96 | 88 | 0.008 | 0.011 |
| 10 | Antigen processing & presentation | Down | 1.55 | 71 | 0.020 | 0.028 |
| 11 | PD-L1 expression and PD-1 checkpoint pathway in cancer | Down | 1.47 | 41 | 0.024 | 0.034 |
| 12 | Interferon-γ response | Down | 1.38 | 66 | 0.029 | 0.042 |
| 13 | NK cell–mediated cytotoxicity | Down | 1.31 | 73 | 0.034 | 0.049 |
